# Understanding the transmission dynamics of *Escherichia coli* O157:H7 super-shedding infections in feedlot cattle

**DOI:** 10.7717/peerj.12524

**Published:** 2021-12-20

**Authors:** Elizabeth M. Antaki-Zukoski, Xunde Li, Bruce Hoar, John M. Adaska, Barbara A. Byrne, Edward R. Atwill

**Affiliations:** 1 Department of Population Health and Reproduction, University of California, Davis, Davis, California, USA; 2 Western Institute for Food Safety and Security, University of California, Davis, California, USA; 3 College of Agriculture and Natural Resources, University of Wyoming, Laramie, Wyoming, USA; 4 Department of Pathology, Microbiology, and Immunology, University of California, Davis, Davis, California, USA; 5 California Animal Health and Food Safety Laboratory, Tulare Branch, University of California, Tulare, California, USA

**Keywords:** *Escherichia coli* O157:H7, Feedlot cattle, Super-shedders, Strain, Ingested dose, Bio-burden

## Abstract

**Background:**

The presence of *Escherichia coli* O157:H7 (*E. coli* O157:H7) super-shedding cattle in feedlots has the potential to increase the overall number (bio-burden) of *E. coli* O157:H7 in the environment. It is important to identify factors to reduce the bio-burden of *E. coli* O157 in feedlots by clarifying practices associated with the occurrence of super-shedders in feedlot cattle.

**Methods:**

The objective of this study is to (1) identify host, pathogen, and management risk factors associated with naturally infected feedlot cattle excreting high concentrations of *E. coli* O157:H7 in their feces and (2) to determine whether the ingested dose or the specific strain of *E. coli* O157:H7 influences a super-shedder infection within experimentally inoculated feedlot cattle. To address this, (1) pen floor fecal samples and herd parameters were collected from four feedlots over a 9-month period, then (2) 6 strains of *E. coli* O157:H7, 3 strains isolated from normal shedder steers and 3 strains isolated from super-shedder steers, were inoculated into 30 one-year-old feedlot steers. Five steers were assigned to each *E. coli* O157:H7 strain group and inoculated with targeted numbers of 10^2^, 10^4^, 10^6^, 10^8^, and 10^10^ CFU of bacteria respectively.

**Results:**

In the feedlots, prevalence of infection with *E. coli* O157:H7 for the 890 fecal samples collected was 22.4%, with individual pen prevalence ranging from 0% to 90% and individual feedlot prevalence ranging from 8.4% to 30.2%. Three samples had *E. coli* O157:H7 levels greater than 10^4^ MPN/g feces, thereby meeting the definition of super-shedder. Lower body weight at entry to the feedlot and higher daily maximum ambient temperature were associated with increased odds of a sample testing positive for *E. coli* O157:H7. In the experimental inoculation trial, the duration and total environmental shedding load of *E. coli* O157:H7 suggests that the time post-inoculation and the dose of inoculated *E. coli* O157:H7 are important while the *E. coli* O157:H7 strain and shedding characteristic (normal or super-shedder) are not.

**Discussion:**

Under the conditions of this experiment, super-shedding appears to be the result of cattle ingesting a high dose of any strain of *E. coli* O157:H7. Therefore strategies that minimize exposure to large numbers of *E. coli* O157:H7 should be beneficial against the super-shedding of *E. coli* O157:H7 in feedlots.

## Introduction

Enterohemorrhagic *Escherichia coli* have been a continuous threat to public health worldwide. This pathogen is associated with clinical symptoms like hemorrhagic colitis and hemolytic uremic syndrome (HUS), which could lead to death. The most common serotype for these cases is O157:H7, which the United States recognized as a major human pathogen in 1982 (Caprioli et al., 2005; Kulow et al., 2012) and accounts for up to 80,000 yearly infections (Katani et al., 2021). Most clusters of human disease are traced to contaminated food or water, but person-to-person transmission can also occur. Cattle are considered a major reservoir of *Escherichia coli* O157:H7 (*E. coli* O157:H7) and other serotypes since contaminated beef has been the source of several large outbreaks of disease (Karmali, 1989; Griffin & Tauxe, 1991).

It has been shown that asymptomatic cattle shed *E. coli* O157:H7 intermittently and at relatively low concentrations, but some individuals can shed high concentrations (≥10^4^ CFU/g of feces) and these animals are termed super-shedders (Xu et al., 2014; Munns et al., 2015; Dixon et al., 2020). Additionally, animals shedding *E. coli* O157:H7 at higher concentrations are thought to transmit the bacterium to a greater number of animals than animals shedding the organism at concentrations less than 10^4^ CFU/g of feces (Fox et al., 2007). According to Stanford, Agopsowicz & McAllister (2012), less than 10% of the feedlot cattle are considered super-shedders for *E. coli* O157:H7, but these animals are considered a contributing factor for sustaining the bacterium within the pen population. This idea is also supported by Omisakin et al. (2003), where it was thought that greater than 96% of *E. coli* O157:H7 isolates within an animal population originated from 9% of super-shedder animals. Fecal shedding of *E. coli* O157:H7 is often seasonal, with increased shedding during the summer, which corresponds to an increased incidence of human disease (Hancock et al., 1994; Chapman et al., 1997). Frequently individual cattle are transiently colonized (<1 month) by one particular strain, but occasionally animals excrete multiple strains of *E. coli* O157:H7. Individual strains of *E. coli* O157:H7 have been isolated from herds for as long as 2 years, whereas other herds can remain culture negative for several years (Besser et al., 1997; Shere, Bartlett & Kasper, 1998).

Many common factors can influence the transmission and prevalence of *E. coli* O157:H7 in cattle, such as age, stress, season, and diet. Management practices can also play a major role in the survival of these pathogens among cattle, potentially supporting a supper-shedder strain within the animal population (Kulow et al., 2012). For example, *E. coli* O157:H7-challenged cattle respond with heterogeneous fecal-shedding periods, where the duration and magnitude are related to the exposure dose and age (Cray & Moon, 1995; Besser et al., 2001). Neonatal calves are the age class susceptible to *E. coli* O157:H7. Related clinical signs include diarrhea, intestinal hyperemia, fibrinous exudates, with pathological attaching/effacing (A/E) lesions in the distal part of the small intestine and entire large intestine. Older calves can have transient watery diarrhea, but intestinal tissue is not seriously affected with the development of A/E lesions. In contrast to calves, *E. coli* O157:H7 challenged cattle older than 12 months lack clinical signs and may or may not have intestinal lesions. When A/E lesions are seen in this older group of animals, they have only been identified in the lymphoid follicle-associated epithelium in the rectoanal junction of both naturally and experimentally infected cattle (Naylor et al., 2003; Lim et al., 2007; Baines, Lee & McAllister, 2008). As a result, the rectoanal junction is hypothesized to be the primary *E. coli* O157:H7 colonization site responsible for persistent shedding in cattle. Katani et al. (2021) suggests that super-shedder strains exhibit a non-locus enterocyte effacement (LEE) strong adherence phenotype to bovine rectoantal junction epithelial cells that may be different when compared to other *E. coli* O157:H7 isolates.

The presence of *E. coli* O157:H7 super-shedding cattle in feedlots have the potential to increase the overall number (bio-burden) of *E. coli* O157:H7 in the environment. Due to this it’s important to identify practices that are associated with this phenomenon in order to reduce the bacterial shedding and transmission between animals. Our objectives were to determine (1) the rate of occurrence and to identify host, pathogen, and management risk factors associated with naturally infected cattle shedding high levels of *E. coli* O157:H7 in their feces and (2) if the ingested dose or a strain type of *E. coli* O157:H7 results in a super-shedder infection in experimentally inoculated cattle.

## Materials and methods

### Enrolling feedlots and sampling

Samples were collected from four feedlots, two in California and two in Texas. Feedlots were chosen based on their willingness to participate, but were representative of typical feeding operations of the region. Two cattle pens, approximately 100 animals, were sampled monthly in each feedlot. One pen had been on feed for less than 30 days (range = 4–30) and the other pen on feed for more than 30 days (range = 42–233). A total of 20 samples were collected from each pen. Fresh feces were collected from the floor of the feedlot pen and placed in a sterile cup (Fisher Scientific, Santa Clara, CA, USA). Sampling occurred over the period of 9 months, starting from September 2011 and ending May 2012. A standard questionnaire was administered at each visit to record information such as total number of animals within the pen, animal age, weight, animal arrival date, health status, pen condition, dietary history, and other related management factors (water source, square footage of pen, pen cover, water cover, type of floor, cleaning schedule). Climate parameters were recorded from nearby weather stations (National Climate Data Center (NCDC), 2011–2012, http://www.ncdc.noaa.gov; California Irrigation Management Information System (CIMIS), 2011–2012, http://www.cimis.water.ca.gov).

### Analytical assays for detecting *E. coli* O157:H7

Fecal samples were placed on ice and returned to the laboratory within 24 h for processing. Ten grams of each sample was enriched in 90 mL of Tryptic Soy Broth (TSB) (Sigma-Aldrich, St. Louis, MO, USA) and incubated for 2 h at 37 °C followed by 8 h at 42 °C. This was followed by Immuno Magnetic Separation (IMS) using anti-O157 antibodies (Dynal Inc., Camarillo, CA, USA) and cultured on CT-SMAC (Becton, Dickinson, Co., Sparks, MD, USA), MacConkey agar with sorbitol, cefixime, and potassium tellurite, and Rainbow agar (Biolog, Hayward, CA, USA) for the presence of *E. coli* O157:H7 (Fox et al., 2007). If colonies were considered O157:H7 suspects, conventional PCR (Eppendorf, Hauppauge, NY, USA) was performed using primers to detect the *rfbE* O-antigen synthesis gene in *E. coli* O157:H7 (Philpott & Ebel, 2003, Chapters 1 and 4). Confirmed colonies were stored at −80 °C in Microbank vials (Pro-lab Diagnostics, Round Rock, TX, USA) for further analysis.

### Quantification of *E. coli* O157:H7

If the above fecal sample was positive, 2 additional 10 g samples were weighed and placed into 90 mL TSB bags. Three 1 g samples were also weighed out and placed in 9 mL of TSB then serially diluted out to 10^−8^ in 9 mL TSB. All additional broth cultures from the positive fecal sample were incubated at 25 °C for 2 h followed by 42 °C for 8 h, shaking at 50 rpm, and held overnight at 6 °C. If the additional 10 g sample bags were positive for the presence of *E. coli* O157:H7, then further IMS and traditional PCR were performed on each 1 g sample with the corresponding dilutions until the samples were negative. Based upon the number of well samples that were positive for *E. coli* O157:H7 in each dilution, the most probable number (MPN) values were determined using United States Food and Drug Administration’s MPN-Bacteriological Analytical Manual computer program. Concentrations were expressed as MPN per gram of feces (Fox et al., 2007). As performed in our laboratory, this IMS and MPN method has been shown to detect as few as 1 MPN/10 g of steer feces (Data not shown).

### Bacterial strain selection for experimental steer inoculation

Six *E. coli* O157:H7 strains were selected from feedlot steers that had originally been sampled in California and Texas (from objective 1). Three strains were defined as normal shedders, isolated from cattle with concentrations of <10^4^ MPN/g of feces and three were defined as super-shedders, isolated from cattle with concentrations of ≥10^4^ MPN/g of feces. Each selected strain was also confirmed by Real-time PCR (Realplex 2.2; Eppendorf, Hauppauge, New York, USA) to contain at least one or both Shiga toxin genes (Philpott & Ebel, 2003, Chapters 1 and 4).

### DNA-fingerprinting of *E. coli* O157:H7 strains

A comparison of *E. coli* O157:H7 strains isolated from super-shedder cattle was made against strains isolated from normal-shedding cattle using the Centers for Disease Control and Prevention standard PulseNet Pulsed Field Get Electrophoresis (PFGE) with X*ba*I restriction enzyme run on a CHEF Mapper (Bio-Rad Laboratories, Hercules, CA, USA) (Ribot et al., 2006). A dendogram was constructed by analyzing each digest profile with band matching and phylogenetic clustering analysis methods in Gel ComparII (Applied Maths, Sint-Martens-Laterm, Belgium) computer software.

### Animal experiments

All animal experiments were conducted under the approval by UC Davis Institutional Animal Care and Use Committee (IACUC) (AUP# 16677). Thirty one, 1-year-old Polled Hereford steers were purchased from the UC Davis campus feedlot. Weights ranged from 795 to 1,135 lbs. Upon arrival, fecal samples were collected per rectum from each steer for three consecutive days. One 10 g sample from each steer was pre-enriched and tested for the presence of *E. coli* O157:H7 as previously described. Steers that were confirmed negative for *E. coli* O157:H7 were transported to the UC Davis Teaching and Research Animal Care Services (TRACS) and housed separately in individual pens inside a biosafety level-II facility. The walls and floor of each pen were cleaned and disinfected with a mixture of bleach and quatracide (Aceland, Mesquite, NV, USA) daily in the early morning. Steers were fed a standard feedlot finishing ration (62% corn, 18% distillers’ grain, 8% alfalfa hay, 4% oat hay, 4% molasses, with monensin sodium) at a rate of approximately 3% BW per day.

### Inoculum preparation and steer inoculation

For each of the six selected *E. coli* O157:H7 strains, growth curves were generated based on profiles of three separate growth trials. A bead of stock was inoculated in 125 mL of TSB and incubated in a shaking incubator (65 rpm) for 6 h at 37 °C, during which time optical density (OD) was checked every 30 min with a spectrophotometer (Shimadzu, Torrance, CA, USA) set at 610 nm wavelength. Serial dilutions were then plated, incubated and original concentrations were calculated as described by Antaki-Zukoski et al. (2019). On the day of inoculation, bacteria were cultured as mentioned previously and five inoculates were prepared for each strain at 10^2^, 10^4^, 10^6^, 10^8^, 10^10^ CFU/mL as determined by OD values. Serial dilutions from 10^−1^ to 10^−10^ were made in 9 mL of PBS and plated in triplicate on LB for each inoculate and incubated overnight at 37 °C. The actual concentration of each inoculate was determined by enumeration of bacterial colonies on plates.

Five steers were assigned to each *E. coli* O157:H7 strain group and inoculated with 10^2^, 10^4^, 10^6^, 10^8^, and 10^10^ CFU of bacteria respectively. An additional steer was used as a negative control for all inoculated steer groups. Each steer was held in a cattle squeeze chute and orally inoculated with gelatin capsules containing one of the five targeted concentrations of bacteria. The oral inoculation was conducted by using a Calf and Colt Balling Gun (Ideal Instruments, Schiller Park, IL, USA).

### Sampling and detection for *E. coli* O157:H7

Feces from each steer were sampled at 24 and 72 h post-inoculation (PI). After this initial time period, the steers were sampled 3 times a week (Monday, Wednesday, and Friday) for roughly 10 weeks. On the morning of sampling days, feces were collected from freshly defecated fecal pats following daily cleaning and disinfection of the pen floors. The surface of each fecal pat was wiped away and approximately 50 g of feces was collected from the middle of the pat with sterile fecal cups (Fisher Scientific, Santa Clara, CA, USA). For each fecal sample, three 10 g samples were placed in 90 mL of TSB and three 1 g samples were placed in 9 mL of TSB. All broth cultures were enriched and tested for the presence of *E. coli* O157:H7 as described previously. Positive samples were then quantified using the same MPN method. Two isolates from each positive steer were then placed in microbank vials and stored at −80 °C.

### Verification of shed bacteria

Pulsed-field gel electrophoresis (PFGE) was used to confirm *E. coli* O157:H7 shed in feces PI was the same strain as the inoculated bacteria. Two isolates from each positive steer were subjected to PFGE. One isolate was chosen from early in the shedding period and one from late in the shedding period in order to confirm persistence of the challenge strain within a given animal. Each isolate was digested with *Xba*I and run alongside the original inoculum strain of the animal to facilitate comparison of the two isolates within the gel. In addition, each late isolate and its original *E. coli* O157:H7 inoculum strain was tested for Shiga toxins 1 and 2, hemolysin A (*hlyA*), and attaching/effacing (*eaeA*) genes (Philpott & Ebel, 2003, Chapters 1 and 4).

### Euthanasia and necropsy

Once a steer was negative for the presence of *E. coli* O157:H7 for three consecutive collection days, it was euthanized with pentobarbital sodium (Euthasol solution, Virbac Animal Health, Fort Worth, Texas) intravenously and necropsied. Steers that remained positive for the presence of *E. coli* O157:H7 throughout the trial were euthanized at approximately 74 days (range = 13 days to 74 days) PI. Sections of the rumen, abomasum, duodenum, proximal jejunum, middle jejunum, distal jejunum, proximal ileum, middle ileum, distal ileum, cecum, spiral colon, proximal colon, distal colon, rectum, liver, mesenteric lymph node, gall bladder, lung, kidney, pancreas were collected and placed in 10% neutral buffered formalin. All tissues were fixed in formalin for a minimum of 2 days before trimming and staining with hemotoxylin and eosin. Histopathology slides were examined for pathological changes in each of the tissue segments and lesions were scored based on intensity. The percentages of total area infected in intestinal sections were determined by counting ten glands in each tissue and calculating the ratio of *E. coli* O157:H7 infected glands to non-infected. Crypt lengths were measured for the proximal colon and distal colon tissue segments at ten different glands and then averaged to compare to the negative control steer gland lengths.

Sections of jejunum, ileum, cecum, spiral colon, and rectum were also collected for culture. Each tissue segment was trimmed into three 10 g pieces and placed into three bags containing 90 mL of TSB. The bags were then placed in a stomacher (400 Circulator; Seward, UK) and homogenized for 1 min at 230 rpm. One milliliter of supernatant from each bag was added to 9 mL of TSB and serial diluted in triplicate as described for the fecal sampling and detection. MPN values were calculated and expressed as MPN per g of tissue.

### Data analysis

The prevalence of *E. coli* O157:H7 in feces was calculated for both pen and feedlot. Chi-square tests of independence were used to assess possible significant relationships. A linear regression was also performed, relating pen-level prevalence to ambient maximum temperature during the 30 days prior to sample collection. Univariable logistic regression was used to identify possible factors (herd size, sex, age, breed, weight, animal arrival date, health status, pen conditions, dietary history, feedlot management practices, and climate factors) associated with the presence of *E. coli* O157:H7 in feces using STATA 11 computer software (StataCorp LP, College Station, TX, USA) (Hoar et al., 2009). Variables that were entered into the model building process had a Pearson’s chi-square statistic *p* ≤ 0.25. Two-way interaction variables were also created from this list and were added into the model. The logistic regression model was built on a forward stepping approach. Variables that had *p* ≤ 0.05 remained in the final statistical model. Host, herd management, and bacterial strain of *E. coli* O157:H7 were possible risk factors, where bacterial shedding status was the outcome variable.

Bacterial counts from daily fecal collections were converted to total environmental shedding load for each steer by multiplying daily MPN counts with the starting weight of the steer and the standard amount of feces produced by one-year-old feedlot steers of similar weight (American Society of Agricultural & Biological Engineers, 2012). The total environmental shedding load was standardized by adding 1 and converted to log_10_ (units = log_10_ (MPN/g of feces)). Colonized bacterial counts (MPN/gram of tissue) in each tissue segment were also standardized by adding 1 and converted to log_10_ (units = log_10_ (MPN/g of tissue)). The effects of time, dose, strain of *E. coli* O157:H7, shedder characteristic of the inoculum (*i.e*. high or normal shedding strain), and starting weight of each steer on the magnitude of daily shedding of *E. coli* O157:H7 was analyzed with Poisson regression performed in STATA 12 computer software (Enemark et al., 2003; La Ragione et al., 2006), with the steer being the random-effect. One-way ANOVA tests were performed with Minitab 15 (Minitab Inc., State College, PA, USA) computer software on mean crypt lengths from ten measured glands in the proximal and distal colon of each steer. Additional *post hoc* tests with Tukey’s multiple-comparison procedure and nonparametric Bonferroni (Dunn) were performed in order to compare each mean against the negative control mean crypt lengths.

## Results

A total of 890 fecal samples were collected and processed from the 4 feedlots. Of this total, 199 (22.4%, 95% CI [15.1% to 29.9%]) were found to be positive for the presence of *E. coli* O157:H7, with 72.1% of the pens having at least one positive fecal sample. The prevalence varied by pen from 0% to 90% and by feedlot from 8.4% to 30.2% (*p* < 0.001). Steers on feed for less than 30 days had a higher percentage (33.2%) of fecal samples that were positive for *E. coli* O157:H7 compared to steers on feed for more than 30 days (16.3%). In the univariable analysis, several management and animal risk factors were associated with the presence of *E. coli* O157:H7, but after a forward stepping model, only the weight at entry to the feedlot and higher daily maximum temperature 30 days prior to sampling remained significant. The final model results are represented in Table 1.

**Table 1:** Results from the final logistic regression model for the presence of *Escherichia coli* O157:H7 in fecal samples from naturally infected feedlot cattle.

Variable	Odds ratio	95% CI	*P*-value
Intercept	0.001		<0.001
Average maximum temperature during the 30 days prior to sampling	1.096	[1.071–1.121]	<0.001
Initial weight at entry to the feedlot	0.998	[0.997–0.999]	<0.001

Most probable numbers of bacteria shed per gram of feces were determined for all but 4 of the 199 positive samples. Table 2 shows a breakdown of these values. Out of these positive samples, 87.6% of them had fewer than 100 MPN/g of feces. Two (1.0%) fecal samples had values greater than 10^4^ MPN/g of feces, classifying them as super-shedders. Another 4 (2.1%) fecal samples had MPN values between 10^3^ and 10^4^/g of feces, with one isolate having a value of exactly 10^4^ MPN/g of feces, also classifying it as a super-shedder. The two super-shedders that were >10^4^ MPN/g of feces were sampled from different feedlots during different sampling events and were from pens where the steers had been on feed for <30 days. Since there were so few samples that were considered super-shedders, regression analysis to determine risk factors associated with their presence was unable to be performed.

**Table 2:** Most probable numbers of bacteria per gram of feces from naturally infected feedlot cattle positive for *Escherichia coli* O157:H7.

Range of MPN/g of feces	Number of samples in range (%)
0–0.1	40 (20.5)
0.1–1	47 (24.1)
1–10	58 (29.7)
10–100	26 (13.3)
100–1,000	18 (9.2)
1,000–10,000	4 (2.1)
10,000–100,000	2 (1.0)
Total	195

**Figure 1:**
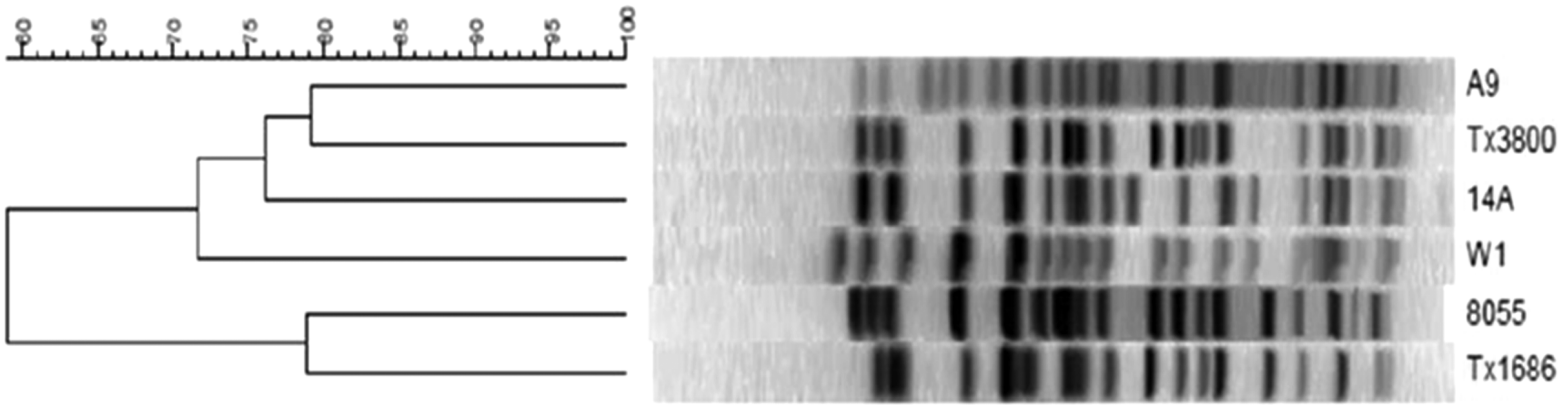
Dendogram of the PFGE anaylsis on the three normal and three super-shedder *Escherichia coli* O157:H7 strains. Normal shedder strains are the A9, W1, 8055 isolates; Super-shedder strains are the Tx3800, 14A, Tx1686 isolates.

The six *E. coli* O157:H7 strains used for the experimental objective were characterized by PFGE prior to inoculation to ensure that each strain was unique. Each of the six strains had a distinct pattern of bands and all six occurred within different groups of feedlot steers. Strain A9 had the most distinct PFGE pattern. This strain had a normal-shedding characteristic isolated from a Texas feedlot, but the pattern didn’t match any other isolates collected at that particular feedlot or any California feedlot (Fig. 1). Real-time PCR on the normal shedder and super-shedder strains demonstrated that all carried Shiga-toxin 2 (*Stx* 2), but only two strains, normal shedder W1 and super-shedder 14A, additionally carried Shiga-toxin 1 (*Stx* 1). Conventional PCR was used to determine that the six inoculum strains carried both attaching/effacing and hemolysin A genes (Table 3). Growth patterns in culture were similar among the six *E. coli* O157:H7 inoculum strains, despite the normal or super-shedder characteristics.

**Table 3:** Normal and super-shedder *Escherichia coli* O157:H7 strains used to inoculate the experimental steers.

Strain ID	Location (State) collected	Date collected	Type of shedding	Concentrations in feces (MPN/g)	Toxins
A9	TX	9/27/2011	Normal	43	Stx 2
W1	CA	9/13/2011	Normal	23	Stx 1/Stx 2
8055	CA	3/21/2012	Normal	2.42	Stx 2
14A	CA	1/18/2012	Super	2.1 × 10^4^	Stx 1/Stx 2
Tx3800	TX	1/27/2012	Super	3.9 × 10^5^	Stx 2
Tx1686	TX	1/27/2012	Super	4.7 × 10^4^	Stx 2

**Figure 2:**
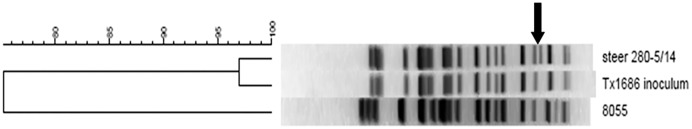
Dendogram of PFGE analysis on the *Escherichia coli* O157:H7 Tx1686 original inoculum, late isolate and the normal shedder 8055 strain. Steer 280-5/14 is the late Tx1686 isolate that has the extra band towards the bottom of the PFGE pattern; the arrow indicates where the extra band is in comparison to the original Tx1686 inoculum strain.

Pulsed-field gel electrophoresis was performed on one early and one late fecal isolate from each positive steer. All isolates were the same as the original *E. coli* O157:H7 inoculum strain, except for the late isolate from the steer inoculated with strain Tx1686 at 10^6^ CFU. This isolate had an additional band on its PFGE pattern in comparison with its original inoculum strain. Since the last normal shedder strain, 8055, was isolated from the same California feedlot where the steers were originally housed before the experiment, a dendogram comparison between the 8055 strain, Tx1686 strain, and the Tx1686 late isolate was performed. The dendogram showed that the unique band pattern of the late Tx1686 isolate was 97% similar to its original inoculum strain rather than the 8055 strain that was isolated from the California feedlot (Fig. 2). In addition to having an extra band in the PFGE pattern on its late *E. coli* O157:H7 isolate, the steer inoculated with strain Tx1686 at 10^6^ CFU was negative for the Shiga-toxin 2 gene after 4 weeks PI, but continued to be positive for the attaching/effacing and hemolysin A genes. Up until that time, the *E. coli* O157:H7 isolates from this steer were positive for Shiga-toxin 2, as was the Tx1686 inoculum strain. All other late isolates contained similar virulence gene patterns in comparison to their original *E. coli* O157:H7 inoculum strains.

In order to account for transient passage of the inoculated bacteria through the gastrointestinal tract, an infected steer was defined as one that shed the inoculated *E. coli* O157:H7 strain for more than 48 h PI. None of the inoculated steers developed clinical signs of infection during the study, except for 1–2 days of diarrhea directly after inoculation. Fecal and selected intestinal tissues were confirmed positive for the presence of the inoculated strain of *E. coli* O157:H7 in all experimentally infected steers. The maximum amount of time a positive steer remained in the study was 74 days PI. The negative control steer was maintained for 77 days after initiation of the study and during this time *E. coli* O157:H7 was not detected in its fecal samples nor from intestinal tissues at necropsy.

**Figure 3:**
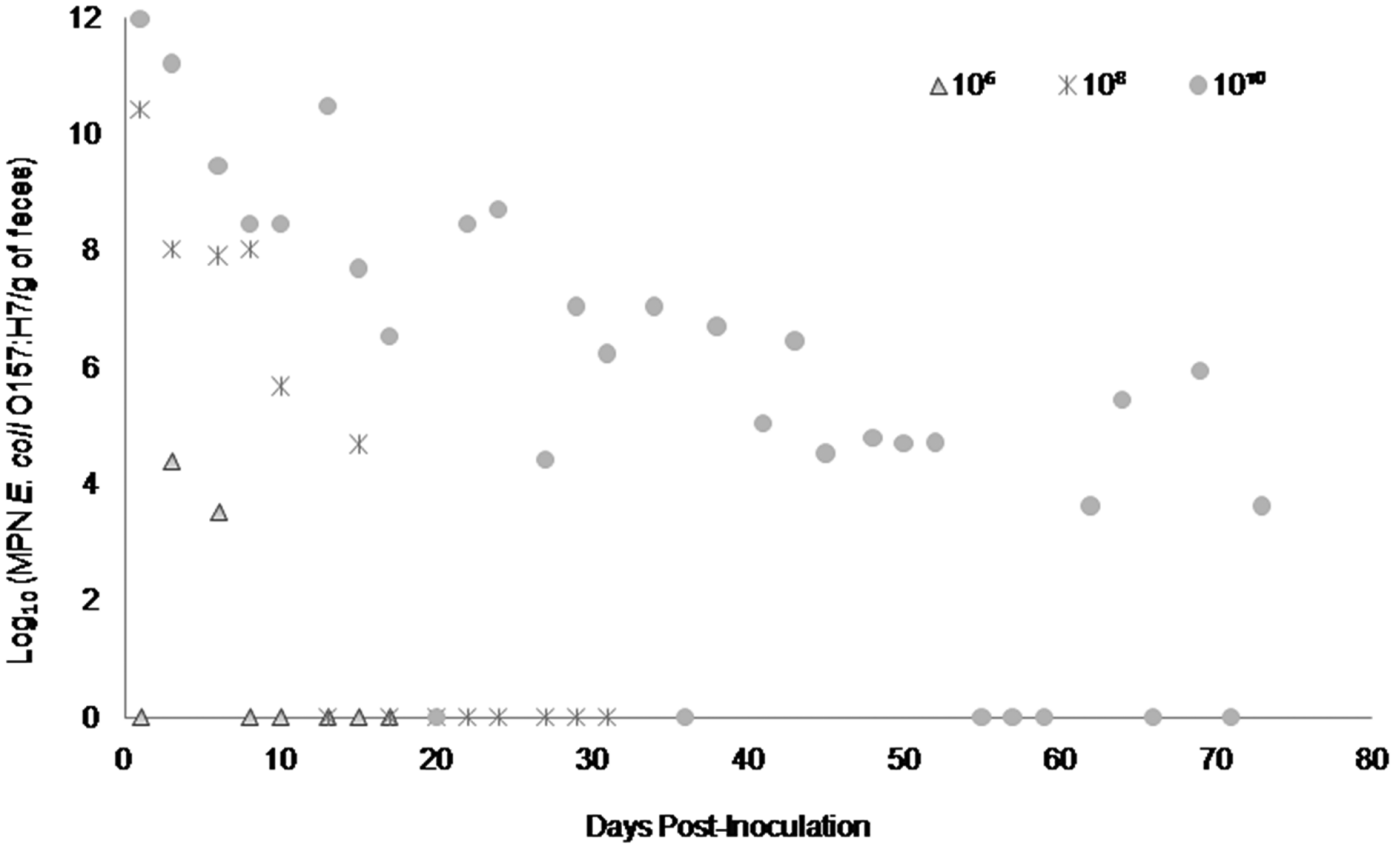
Duration and concentration (Log_10_ (MPN *E. coli* O157:H7/g of feces)) of daily fecal *Escherichia coli* O157:H7 from each steer inoculated with strain A9 (normal shedder inoculum). Steers that did not become infected with their assigned inoculum dose are not shown, these are 10^2^, 10^4^ CFU.

**Figure 4:**
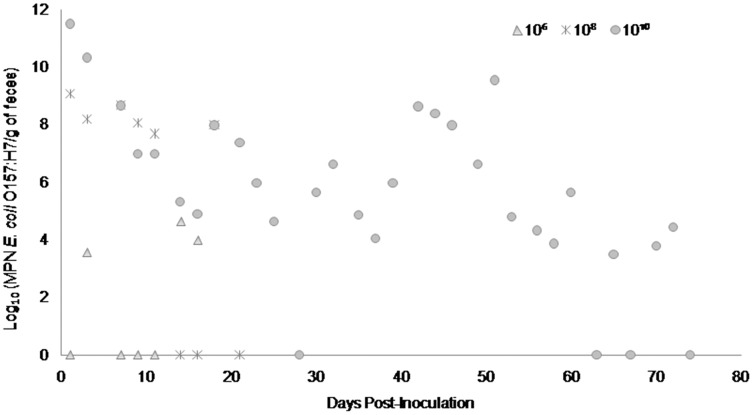
Duration and concentration (Log_10_ (MPN *E. coli* O157:H7/g of feces)) of daily fecal *Escherichia coli* O157:H7 from each steer inoculated with strain W1 (normal shedder inoculum). Steers that did not become infected with their assigned inoculum dose are not shown, these are 10^2^, 10^4^ CFU.

**Figure 5:**
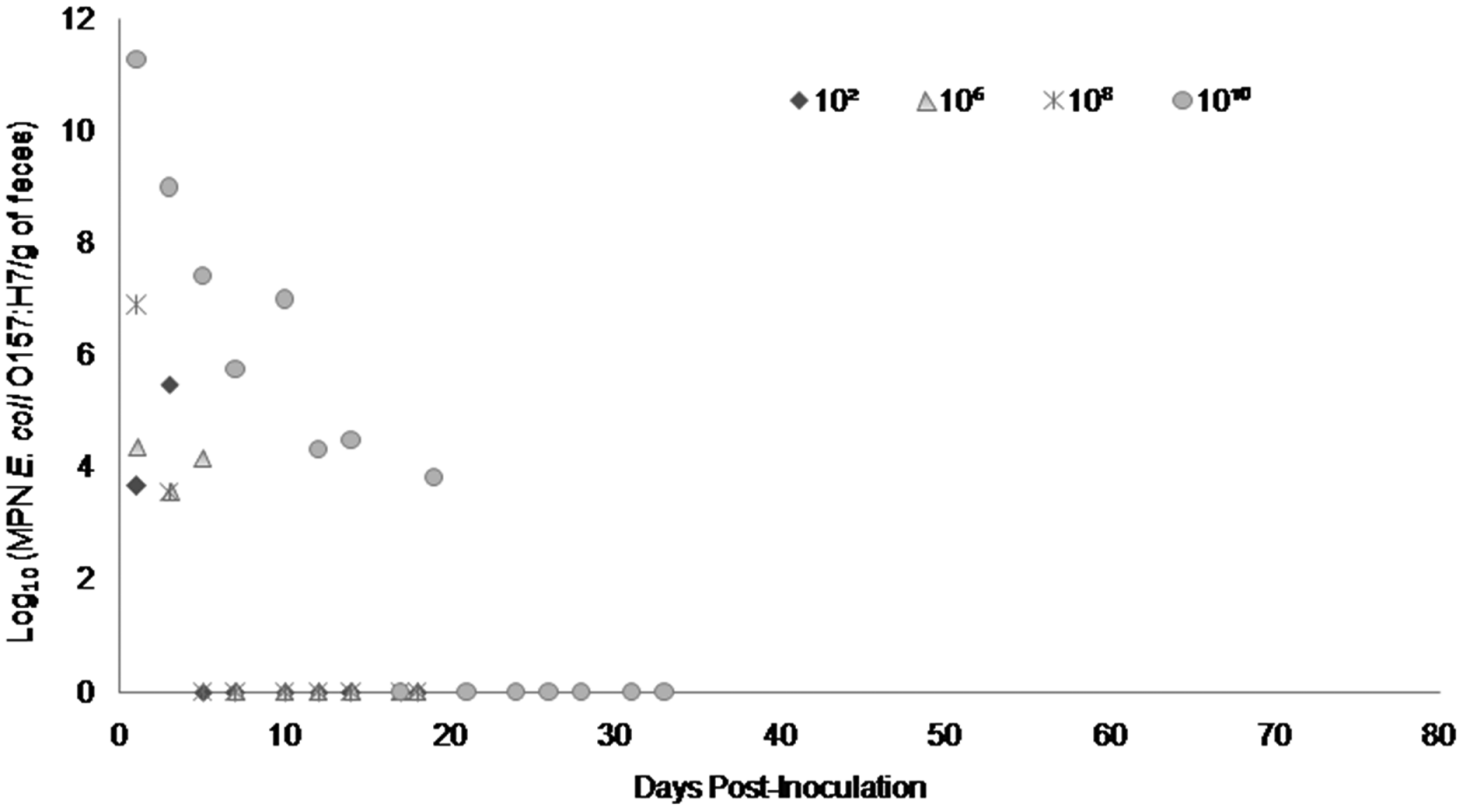
Duration and concentration (Log_10_ (MPN *E. coli* O157:H7/g of feces)) of daily fecal *Escherichia coli* O157:H7 from each steer inoculated with strain 8055 (normal shedder inoculum). Steers that did not become infected with their assigned inoculum dose are not shown, these are 10^4^ CFU.

Fecal shedding of *E. coli* O157:H7 by steers inoculated with normal shedder strains followed a dose-dependent trend with higher doses resulting in shedding for longer periods of time. The 10^10^ CFU inoculated steers for strains A9 and W1 were still shedding the *E. coli* O157:H7 inoculum strain when euthanized at 74 days PI (Figs. 3 and 4). The total environmental shedding load of *E. coli* O157:H7 or log_10_ (MPN/g of feces) gradually decreased in the steer inoculated with strain A9 at 10^10^ CFU, but also had small spikes at 30 and 70 days PI. This shedding pattern is similar to the steer inoculated with strain W1 at 10^10^ CFU which had several spikes of *E. coli* O157:H7 total environmental shedding loads between 20 and 50 days PI. Strain 8055 *E. coli* O157:H7 shedding patterns were much shorter in duration when compared to the other *E. coli* O157:H7 strains with similar shedding behaviors. The 10^10^ CFU inoculum dose of strain 8055 resulted in only 19 days of *E. coli* O157:H7 total environmental shedding PI, with values decreasing over time (Fig. 5).

**Figure 6:**
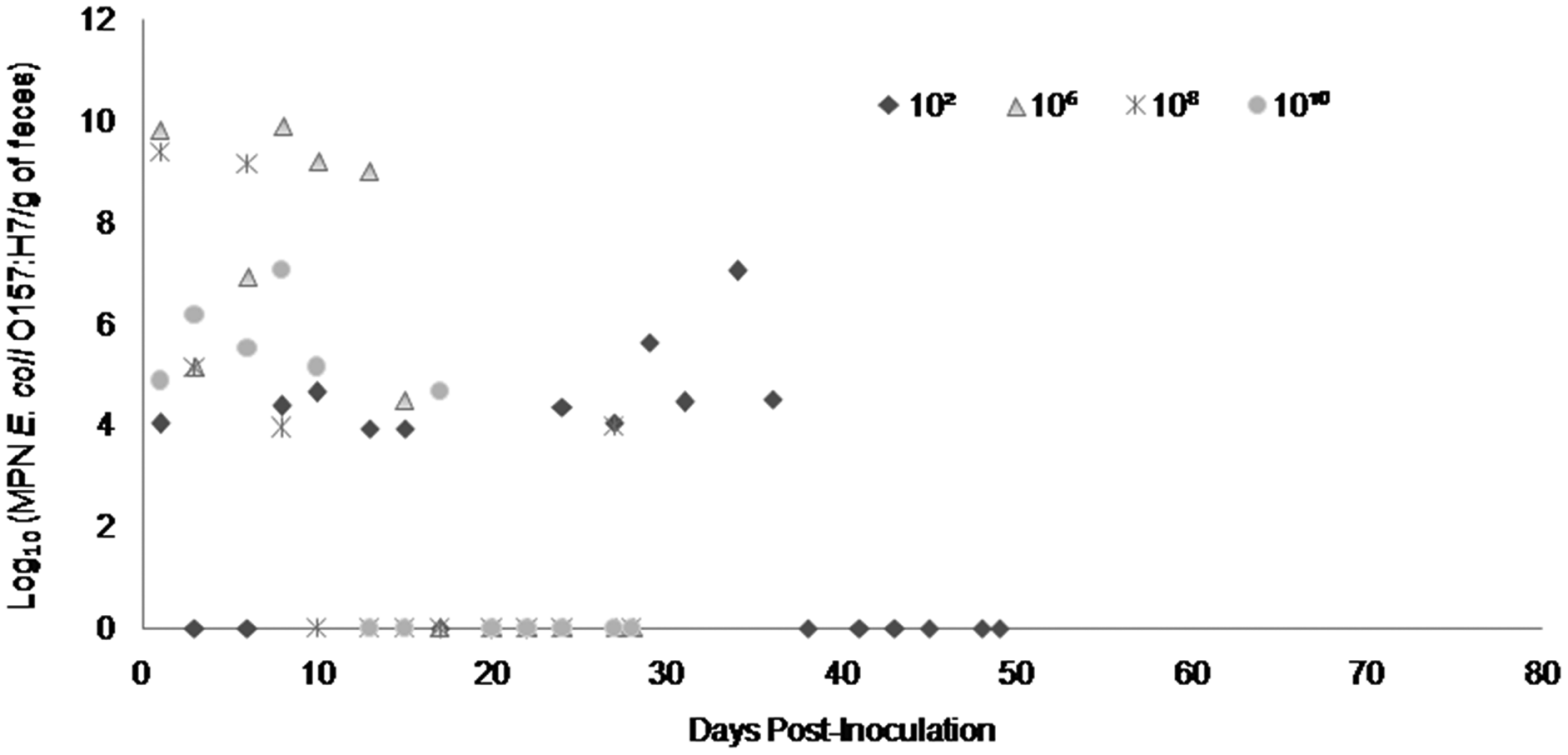
Duration and concentration (Log_10_ (MPN *E. coli* O157:H7/g of feces)) of daily fecal *Escherichia coli* O157:H7 from each steer inoculated with strain Tx3800 (super-shedder inoculum). Steers that did not become infected with their assigned inoculum dose are not shown, these are 10^4^ CFU.

**Figure 7:**
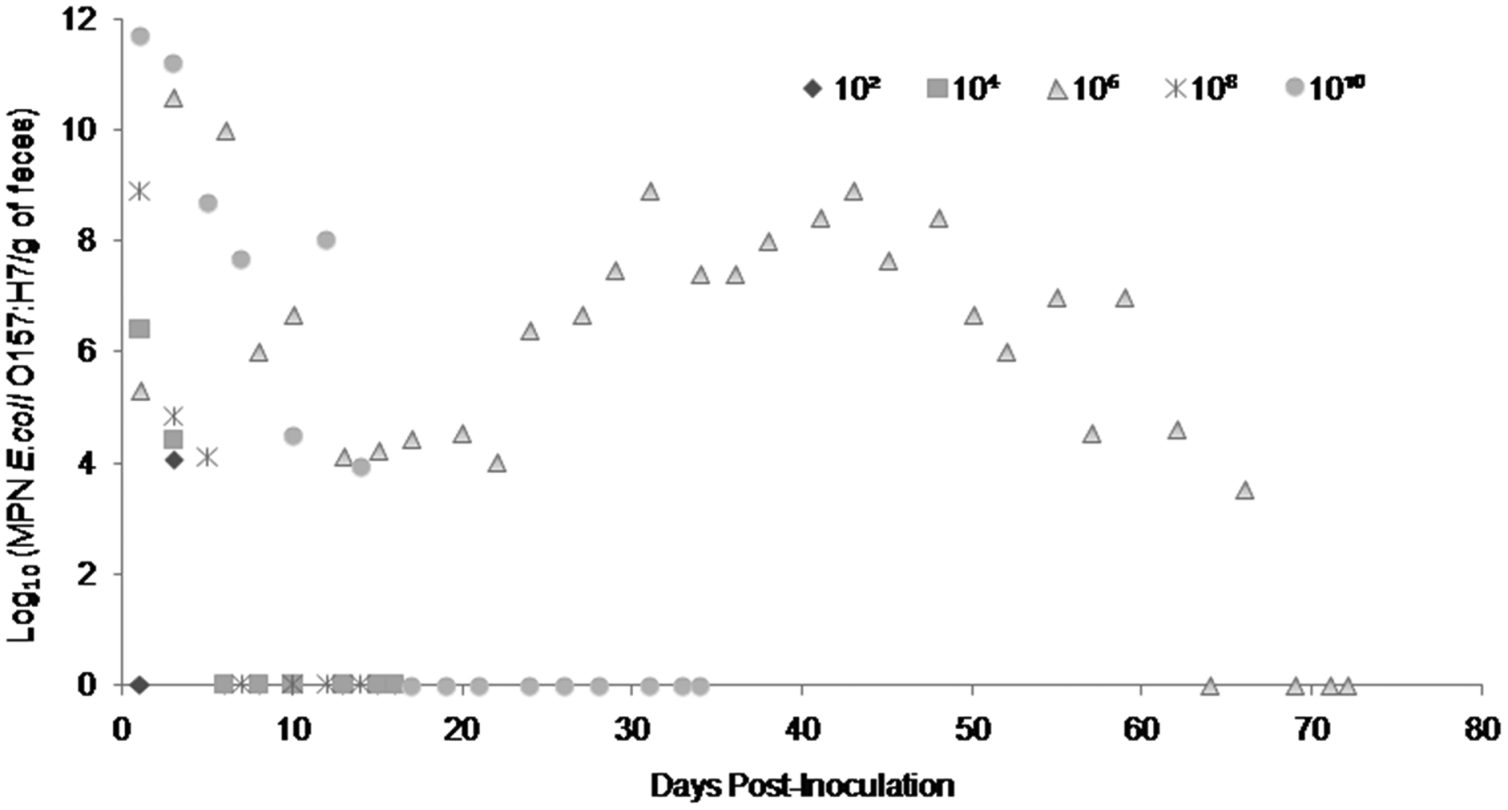
Duration and concentration (Log_10_ (MPN *E. coli* O157:H7/g of feces)) of daily fecal *Escherichia coli* O157:H7 from each steer inoculated with strain Tx1686 (super-shedder inoculum). All steers were considered infected for this inoculum group.

**Figure 8:**
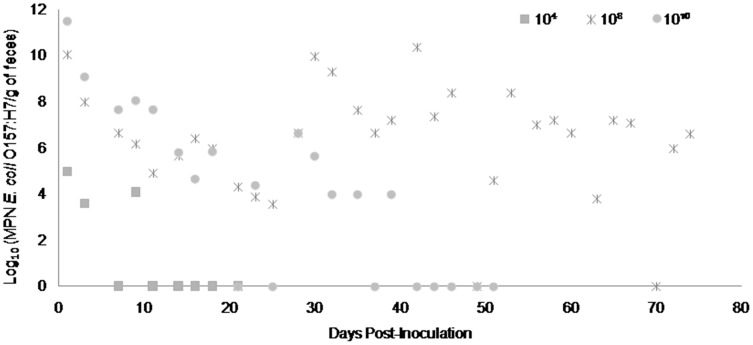
Duration and concentration (Log_10_ (MPN *E. coli* O157:H7/g of feces)) of daily fecal *Escherichia coli* O157:H7 from each steer inoculated with strain 14A (super-shedder inoculum). Steers that did not become infected with their assigned inoculum dose are not shown, these are 10^2^, 10^6^ CFU.

The steers inoculated with super-shedder strains had different shedding patterns in comparison to the normal shedder strains, where high inoculum doses did not always result in longer total environmental shedding of *E. coli* O157:H7. Steers inoculated with strains Tx3800 at 10^2^ CFU and Tx1686 at 10^6^ CFU shed *E. coli* O157:H7 longer than the steers inoculated at larger numbers of the same strain of *E. coli* O157:H7 (Figs. 6 and 7). The steer inoculated at 10^2^ CFU with strain Tx3800 only had one *E. coli* O157:H7 spike at 34 days PI, whereas the rest of the positive *E. coli* O157:H7 samples stayed at lower shedding concentrations. The steer inoculated at 10^6^ CFU of strain Tx1686 produced a bell shaped shedding pattern after a small initial decline in *E. coli* O157:H7 concentrations. Days 30 and 45 PI detected the two highest total environmental shedding load values of *E. coli* O157:H7, while the rest of the *E. coli* O157:H7 concentrations varied in range. Steers inoculated with the 14A strain followed more of a dose-dependent trend, but the steer inoculated at 10^8^ CFU shed *E. coli* O157:H7 for 74 days PI compared to the steer inoculated at 10^10^ CFU which only shed *E. coli* O157:H7 for 39 days PI (Fig. 8). The steer inoculated at 10^8^ CFU with strain 14A had a gradual decline in *E. coli* O157:H7 shedding loads, but at 30 and 45 days PI; the concentrations spiked. In addition to these two spikes in *E. coli* O157:H7 concentrations, this steer continued to shed high environmental loads of *E. coli* O157:H7 between 30 and 74 days PI. Table 4 represents the number of samples where steers from each strain group shed ≥10^4^ total environmental shedding loads and were considered super-shedders.

**Table 4:** Fecal *Escherichia coli* O157:H7 samples that were collected from experimentally inoculated positive steers and were considered super-shedders (concentration of ≥10^4^ total environmental shedding loads).

Type of shedder	Strain ID	Dose of inoculum				
		10^2^	10^4^	10^6^	10^8^	10^10^
Normal	A9	0	0	1	6	23
	W1	0	0	2	6	25
	8055	1	0	2	1	7
Super	14A	0	2	0	27	14
	Tx3800	11	0	7	5	6
	Tx1686	1	2	27	3	7

Statistically, the time PI and the dose of inoculated *E. coli* O157:H7 had a significant effect on the total environmental shedding load of *E. coli* O157:H7 in all steers (*p* ≤ 0.001 and *p* ≤ 0.001), while the shedder characteristic of the inoculum, strain of *E. coli* O157:H7 and starting weight of the steers were non-significant (*p* > 0.05).

At the end of the study, only 3 out of the 30 experimentally inoculated steers had intestinal tissues that were positive for the presence of *E. coli* O157:H7. Steers inoculated with strain W1 at 10^10^ CFU and strain Tx1686 at 10^8^ CFU had one intestinal segment (jejunum and cecum) that was positive for *E. coli* O157:H7 compared to the steer inoculated with strain 14A at 10^8^ CFU which had three tissue segments (cecum, spiral colon, and rectum) that were positive and had higher concentrations of cultured *E. coli* O157:H7. Steers inoculated with strain W1 at 10^10^ CFU and strain 14A at 10^8^ CFU stayed in the study for 74 days whereas the steer inoculated at 10^8^ CFU with strain Tx1686 only stayed in the study for 14 days. On histopathological examination, no intestinal lesions were seen in any of the experimentally inoculated steers. After ANOVA and *post hoc* testing, mean crypt lengths in the proximal and distal colon were not significantly different when compared to the negative control crypt lengths; even though there were several steers with proximal and distal colon mean crypt lengths that were longer than the negative control lengths.

## Discussion

The focus of this work was to investigate the mechanisms by which a small percentage of feedlot cattle become super-shedders. In objective 1 of this study, pen prevalence of *E. coli* O157:H7 in naturally infected feedlot cattle varied from 0% to 90% and feedlot prevalence varied from 8.4% to 30.2%. These results are similar to another cross-sectional study that was performed in 2003 which looked at feedlot prevalence over four states. The study found the individual prevalence to be 10.2%, with 52% of pens and 96% of feedlots having positive *E. coli* O157:H7 samples (Sargeant et al., 2003). Dixon et al. (2020), analyzed 750 fecal samples, collected from 150 feedlot cattle in 10 pens and found that the cumulative animal-level prevalence was estimated at 9.5% for *E. coli* O157:H7. The relationship between increased temperature, lower weight at entry to the feedlot and increased prevalence of *E. coli* O157:H7 has also been discussed by several research groups (Kondo et al., 2010; Williams et al., 2010). There has been some speculation that this seasonal effect is due to day length impacting hormones in cattle (Edrington et al., 2007). Other researchers, like Sheng et al. (2016), think that seasonal differences are due to the exposure dose of the pathogen by the steer coming into contact with infected non-cattle animals or contaminated environmental reservoirs rather than intrinsic or gut microbiota factors. Young animals, particularly cattle, have been extensively studied to show that they are the susceptible age-class for many infections, including *E. coli* O157:H7 (Naylor et al., 2003; Lim et al., 2007; Baines, Lee & McAllister, 2008). Previous studies have demonstrated that the highest prevalence of fecal shedding of *E. coli* O157:H7 in feedlot cattle occurs early in the feeding period, in the weeks immediately following weaning. This can be due to the stress of change, concurrent infections, or dietary changes that occur when young steers enter the feedlot (Laegreid, Elder & Keen, 1999). Henry et al. (2019), thinks that there might be another factor that drives the prevalence of *E. coli* O157:H7 in cattle. The researchers looked at data collected during two cross-sectional surveys in cattle intended for the food chain in Scotland, England, and Wales. Factors associated with the detection of *E. coli* O157:H7 in cattle groups were varied among farms, but herd size was a consistent factor that was identified.

Out of 195 samples enumerated for *E. coli* O157:H7, only two (1.0%) were considered super-shedders. Another 4 (2.1%) fecal samples had MPN values between 10^3^ and 10^4^/g of feces. This low prevalence of super-shedders in feedlot cattle was also seen in a study by Arthur et al. (2013). In that study, 5,086 cattle were analyzed for super-shedding *E. coli* O157:H7. Overall, super-shedders were identified as 2.01% (*n* = 102, CI [1.64 to 2.43%]) of the bovine population tested. The maximum shedding level observed was 4 × 10^7^ CFU/swab (Arthur et al., 2013). This low prevalence of super-shedders is also supported by a study that was conducted by Dixon et al. (2020). Here the researchers found that only 3.3% of the positive cattle shedding Shiga toxin-producing *E.coli* in their feces were considered super-shedders.

In the experimental infections during our study, each inoculum strain used had a different PFGE pattern when compared to one another, but strain A9 was the most unique. This strain was isolated from a Texas feedlot during the month of September and no other isolates sampled from that particular feedlot matched its PFGE pattern. Since the initial concentration when isolated from the feedlot was 43 MPN/g of feces, it is possible that this strain of *E. coli* O157:H7 was overshadowed in the feedlot by a different strain of *E. coli* O157:H7 that was shed in higher amounts (LeJeune et al., 2004; Chase-Topping et al., 2008). As a result, this A9 isolate with the unique PFGE pattern was not seen in later collection dates used for selecting super-shedder inoculum strains. Even though the six inoculum strains had different PFGE patterns, they all carried virulence genes potentially allowing them to attach to the intestinal mucosa with intimin and cause disease when inoculated into a host. All isolates of *E. coli* O157:H7 that were used had at least Stx2, which according to Teng et al., 2020, allows the pathogen to enhance adherence and colonization of intestinal epithelial cells, possibly increasing the expression of the host cell receptor so that initial binding can occur.

No clinical signs were seen in any of the experimentally inoculated steers, except for 1–2 days of diarrhea directly after inoculation. The diarrhea may have been secondary to stress incurred during the inoculation process. Each steer was led into a cattle squeeze chute and orally inoculated while animal handlers and veterinarians examined the health condition of the steers. Stress levels of the steers increased once they were separated and individually housed in stalls where steer-to-steer contact was not possible. It was observed that by 2 days PI, the steers became accustomed to their surroundings and fecal samples went back to normal consistency. The concept that initial onset of diarrhea could be brought on by stress instead of the infection with *E. coli* O157:H7 has also been reported by Cray & Moon (1995) when *E. coli* O157:H7 was experimentally inoculated into calves and adult cattle.

It was determined that the time PI and the dose of inoculated *E. coli* O157:H7 had a significant effect on the total environmental shedding load of *E. coli* O157:H7 in the feces of all steers. It was interesting to see that the shedding characteristic of the inoculum (normal or super-shedder), strain of *E. coli* O157:H7 (a particular strain) and starting weight of the steers were not significant factors on the length and concentration of *E. coli* O157:H7 shed in the feces. Sheng et al. (2016) also demonstrated that the inoculated strain of STEC had no significant effect on colonization and that no single strain was dominant among positive steers. Steers inoculated with the normal shedder strains (A9, W1, 8055) tended to follow a dose-dependent shedding pattern with cattle receiving higher doses shedding for longer periods of time with 1–2 super-shedder concentration spikes of *E. coli* O157:H7. This can be supported by Kulow et al. (2012) where they determined that the inoculum dose has an significant influence on the concentration of *E. coli* O157:H7 shed in the feces. Also reported in the previous study, persistent colonization was detected for the duration of several weeks in some steers either as continuous shedding or shedding patterns interrupted by less than 2 days (Kulow et al., 2012). In our study, the steers inoculated with the super-shedder inoculum strains did not follow this type of dose-dependent *E. coli* O157:H7 shedding trend. Variations in host susceptibility, stress, and intestinal microbiota could account for the differences in fecal *E. coli* O157:H7 shedding patterns between the normal and super-shedder strains (Matthews et al., 2006). Wells et al. (2020), stated that varied results in pathogen survival and the ability to pass through the gastrointestinal tract is related to the potential acid-resistance capability of some *E. coli* O157:H7, resulting in possible super-shedder events. The ability to colonize the abomasum of cattle may play a slight role in the development of *E. coli* O157:H7 super-shedder infections among cattle cohorts. Results have varied, but additional research needs to be conducted.

The steer inoculated with strain Tx1686 at 10^6^ CFU had a slightly different PFGE pattern in comparison with its original inoculum strain and lost its Shiga-toxin 2 gene 4 weeks PI, but continued to be positive for the attaching/effacing and hemolysin A genes. The Shiga-toxins in Shiga-toxin producing *E. coli* are encoded by diverse lambdoid bacteriophages that are highly mobile genetic elements that play an important part in horizontal gene transfer and genome diversification (Johannes & Romer, 2010). Due to these highly mobile genetic elements, the late isolate from the steer inoculated with Tx1686 at 10^6^ CFU lost its Shiga-toxin 2 bacteriophage and possibly gained a genetic element that was not examined for, resulting in the extra band on the PFGE pattern. This phenomenon could be due to multiple host-pathogen interaction factors, but it’s interesting to see that it only occurred in one steer within the *E. coli* O157:H7 inoculum group. Future molecular studies with this isolate could determine the extra genetic material. Additional characterization of the Stx2 subtypes from each *E. coli* O157:H7 strain used in this study could also support this change in virulence factors. Teng et al. (2020), states that Stx2a has a critical role in animal-to-animal transmission, leading to super-shedder infections.

On histopathological examination, no changes consistent with attaching-and-effacing lesions or adherent bacterial layers were seen in any of the sections of the gastrointestinal tract. The lack of evidence of adherent bacteria or attaching-and-effacing lesions in the small or large intestine may be due to low tissue *E. coli* O157:H7 concentrations. The absence of lesions was also seen in Cray & Moon (1995) where tissue *E. coli* O157:H7 concentrations were below 10^6^ CFU/g, which is thought to be the threshold for recognition of adherent layers of bacteria in histologic sections. Another reason for the absence of lesions could be that the steers were euthanized after being negative for three consecutive days PI. Tissue was positive for the presence of *E. coli* O157:H7 in 3 out of the 30 experimental steers, but concentrations were very low. The steer inoculated with strain 14A at 10^8^ CFU had the highest concentrations of *E. coli* O157:H7 and the organism was present in the cecum, spiral colon, and rectum. This steer shed its inoculated *E. coli* O157:H7 strain for 74 days PI and was still culture positive at this time point. The continuous shedding of the *E. coli* O157:H7 strain in high concentrations could account for the wider distribution of culture positive tissues. The longer mean crypt lengths of the experimental steers in comparison with the negative control steer is possibly due to the intestinal mucosa responding to the presence of the *E. coli* O157:H7 pathogen by increasing its numbers of mucosal cells, as seen in Coutinho et al. (2008) and Magnuson et al. (2000). Even though the mean crypt lengths were longer in comparison to the negative control these were not statistically significant.

Due to financial and space restrictions, inoculum doses of each *E. coli* O157:H7 strain was represented by only one steer and the maximum amount of time an *E. coli* O157:H7-positive steer was kept in the study was 74 days PI. These restrictions on the number of steers used for each inoculum dose and the amount of time a positive steer remained in the study, could also account for differences in fecal shedding patterns. Euthanizing the steers while they were still positive for *E. coli* O157:H7 truncated the shedding pattern, preventing determination of maximum shedding duration of the *E. coli* O157:H7 strain. Another limitation was that the experimental study was only conducted in one replication, instead of having several to account for variations in host factors. If additional trials were conducted, then the shedding characteristics of each selected *E. coli* O157:H7 strain could be examined over a longer period of time, providing the ability to compare trial results with each inoculated strain. Even with these limitations, this study was able to show that there are multiple factors (farm management and host) that cause cattle to become a super-shedder of *E. coli* O157:H7, which was supported by using unique *E. coli* O157:H7 strains that were circulating within the environment and inoculating experimental steers at various concentrations to observe fecal shedding characteristics. Strains may be more virulent, but there is no single strain of *E. coli* O157:H7 that is considered a “super-shedder” (Arthur et al., 2013).

## Conclusions

In summary, results from this study demonstrated that the occurrence of super-shedding *E. coli* O157:H7 is low in feedlot cattle and is not the result of infection with unique virulent strains of *E. coli* O157:H7 (super-shedder strains). Instead, super-shedding is the result of cattle ingesting a high dose of any strain of *E. coli* O157:H7 under the same experimental conditions and with the same inoculum strains. With this understanding, further on-farm intervention strategies to reduce individual animal infections can decrease the overall feedlot prevalence, resulting in less bacterial shedding of *E. coli* O157:H7 into the environment.

## Supplemental Information

10.7717/peerj.12524/supp-1 Supplemental Information 1 Dataset for objective 1.

10.7717/peerj.12524/supp-2 Supplemental Information 2 Dataset for objective 2.

10.7717/peerj.12524/supp-3 Supplemental Information 3 Statistical data for objective 1.

10.7717/peerj.12524/supp-4 Supplemental Information 4 Pathology measurements for objective 2.

10.7717/peerj.12524/supp-5 Supplemental Information 5 Raw gel images for fingerprint comparisons. Raw gel images of bacterial fingerprint patterns for dendogram construction.

10.7717/peerj.12524/supp-6 Supplemental Information 6 Feedlot questionnaire.

## Additional Information and Declarations

### Data Availability

The following information was supplied regarding data availability: Raw data are available in the Supplemental Files.

## References

[ref-1] Antaki-Zukoski E, Li X, Pesavento P, Adaska J, Byrne B, Nguyen T, Hoar B, Jay-Russell M, Atwill E (2019). *Cryptosporidium parvum* co-infection on the magnitude of *Escherichia coli* O157: H7 shedding in experimentally inoculated pigs. Jacobs Journal of Veterinary Science and Research.

[ref-2] Arthur T, Ahmed R, Chase-Topping M, Kalchayanand N, Schmidt J, Bono J (2013). Characterization of *Escherichia coli* O157: H7 strains isolated from supershedding cattle. Applied and Environmental Microbiology.

[ref-3] American Society of Agricultural and Biological Engineers (2012). Manure production for beef cattle. http://www.asabe.org.

[ref-4] Baines D, Lee B, McAllister T (2008). Heterogeneity in enterohemorrhagic *Escherichia coli* O157: H7 fecal shedding in cattle is related to *Escherichia coli* O157: H7 colonization of the small and large intestine. Canadian Journal of Microbiology.

[ref-5] Besser T, Hancock D, Pritchett L, McRae E, Rice D, Tarr P (1997). Duration of detection of fecal excretion of *Escherichia coli* O157: H7 in cattle. Journal of Infectious Diseases.

[ref-6] Besser T, Richards B, Rice D, Hancock D (2001). *Escherichia coli* O157: H7 infection of calves: infectious dose and direct contact transmission. Epidemiology and Infection.

[ref-7] Caprioli A, Morabito S, Brugere H, Oswald E (2005). Enterohemorrhagic *Escherichia coli*: emerging issues on virulence and modes of transmission. Veterinary Research.

[ref-8] Chapman P, Siddons C, Cerdan Malo A, Harkins M (1997). A 1-year study of *Escherichia coli* O157 in cattle, sheep, pigs, and poultry. Epidemiology and Infection.

[ref-9] Chase-Topping M, Gally D, Low C, Matthews L, Woolhouse M (2008). Super-shedding and the link between human infection and livestock carriage of *Escherichia coli* O157. Nature.

[ref-10] Coutinho B, Oria R, Vieira C, Sevilleja J, Warren C, Maciel J, Thompson M, Pinkerton R, Lima A, Guerrant R (2008). *Cryptosporidium* infection causes undernutrition and, conversely, weanling undernutrition intensifies infection. Journal of Parasitology.

[ref-11] Cray W, Moon H (1995). Experimental infection of calves and adult cattle with *Escherichia coli* O157: H7. Applied and Environmental Microbiology.

[ref-12] Dixon A, Cernicchiaro N, Amachawadi R, Shi X, Cull C, Renter D (2020). Longitudinal characterization of prevalence and concentration of Shiga toxin-producing *Escherichia coli* serogroups in feces of individual feedlot cattle. Foodborne Pathogens and Disease.

[ref-13] Edrington T, Callaway T, Ives S, Engler M, Looper M, Anderson R, Nisbet D (2007). Seasonal shedding of *Escherichia coli* O157: H7 in ruminants: a new hypothesis. Foodborne Pathogens and Disease.

[ref-14] Enemark H, Bille-Hansen V, Lind P, Heegaard P, Vigre H, Ahrens P, Thamsborg S (2003). Pathogenicity of *Cryptosporidium parvum*-evaluation of an animal infection model. Veterinary Parasitology.

[ref-15] Fox T, Renter D, Sanderson M, Thomson D, Lechtenberg K, Nagaraja T (2007). Evaluation of culture methods to identify bovine feces with high concentrations of *Escherichia coli* O157. Applied and Environmental Microbiology.

[ref-16] Griffin P, Tauxe R (1991). The epidemiology of infections caused by *Escherichia coli* O157: H7, other enterohemorrhagic *E. coli*, and the associated hemolytic-uremic syndrome. Epidemiologic Reviews.

[ref-17] Hancock D, Besser T, Kinsel M, Tarr P, Rice D, Paros M (1994). The prevalence of *Escherichia coli* O157: H7 in dairy and beef in Washington state. Epidemiology and Infection.

[ref-18] Henry M, McCann C, Humphry R, Morgan M, Willett A, Evans J, Gunn G, Tongue S (2019). The British *E. coli* O157 in cattle study (BECS): factors associated with the occurrence of *E. coli* O157 from contemporaneous cross-sectional surveys. BMC Veterinary Research.

[ref-19] Hoar B, Paul R, Siembieda J, das Fracas C, Pereira M, Atwill E (2009). *Giardia duodenalis* in feedlot cattle from the central and western United States. BMC Veterinary Research.

[ref-20] Johannes L, Romer W (2010). Shiga toxins- from cell biology to biomedical applications. Nature.

[ref-21] Karmali M (1989). Infection by verocytotoxin-producing *Escherichia coli*. Clinical Microbiology Reviews.

[ref-23] Katani R, Kudva I, Srinivasan S, Stasko J, Schilling M, Li L, Cote R, DebRoy C, Arthur T, Sokurenko E, Kapur V (2021). Strain and host-cell dependent role of type-1 fimbriae in the adherence phenotype of super-shed *Escherichia coli* O157: H7. International Journal of Medical Microbiology.

[ref-24] Kondo S, Hoar B, Villanueva V, Mandrell R, Atwill E (2010). Longitudinal prevalence and molecular typing of *Escherichia coli* O157: H7 by use of multiple-locus variable-number tandem-repeat analysis and pulsed-field gel electrophoresis in fecal samples collected from a range-based herd of beef cattle in California. American Journal of Veterinary Research.

[ref-25] Kulow M, Gonzales T, Pertzborn K, Dahm J, Miller B, Park D, Gautam R, Kasper C, Ivanek R, Dopfer D (2012). Differences in colonization and shedding patterns after oral challenge of cattle with three *Escherichia coli* O157: H7 strains. Applied and Environmental Microbiology.

[ref-26] Laegreid W, Elder R, Keen J (1999). Prevalence of *Escherichia coli* O157: H7 in range beef calves at weaning. Epidemiology and Infection.

[ref-27] La Ragione R, Best A, Clifford D, Weyer U, Johnson L, Marshall R, Marshall J, Cooley W, Farrelly S, Pearson G, Woodward M (2006). Influence of colostrums deprivation and concurrent *Cryptosporidium* parvum infection on the colonization and persistence of *Escherichia coli* O157: H7 in young lambs. Journal of Medical Microbiology.

[ref-28] LeJeune J, Besser T, Rice D, Berg J, Stilborn R, Hancock D (2004). Longitudinal study of fecal shedding of *Escherichia coli* O157: H7 in feedlot cattle: predominance and persistence of specific clonal types despite massive cattle population turnover. Applied and Environmental Microbiology.

[ref-29] Lim J, Li J, Sheng H, Besser T, Potter K, Hovde C (2007). *Escherichia coli* O157: H7 colonization at the rectoanal junction of long-duration culture positive cattle. Applied and Environmental Microbiology.

[ref-30] Magnuson B, Davis M, Hubele S, Austin P, Kudva I, Williams C, Hunt C, Hovde C (2000). Ruminant gastrointestinal cell proliferation and clearance of *Escherichia coli* O157: H7. Infection and Immunity.

[ref-31] Matthews L, McKendrick I, Ternent H, Gunn G, Synge B, Woolhouse M (2006). Super-shedding cattle and the transmission dynamics of *Escherichia coli* O157. Epidemiology and Infection.

[ref-32] Munns K, Selinger B, Stanford K, Guan L, Callaway T, McAllister T (2015). Perspectives on super-shedding of *Escherichia coli* O157: H7 by cattle. Foodborne Pathogens and Disease.

[ref-33] Naylor S, Low C, Besser T, Mahajan A, Gunn G, Pearce M, McKendrick I, Smith G, Gally D (2003). Lynphoid follicle-dense mucosa at the terminal rectum is the principal site of colonization of Enterohemorrhagic *Escherichia coli* O157: H7 in the bovine host. Infection and Immunity.

[ref-34] Omisakin F, MacRae M, Ogden ID, Strachan NJ (2003). Concentration and prevalence of *Escherichia coli* O157 in cattle feces at slaughter. Applied and Environmental Microbiology.

[ref-35] Philpott D, Ebel F (2003). Chapters 1 and 4: methods in molecular medicine: E. coli-shiga toxin methods and protocols.

[ref-36] Ribot E, Fair M, Gautom R, Cameron D, Hunter S, Swaminathan B, Barrett T (2006). Standardization of pulsed-field gel electrophoresis protocols for the substyping of *Escherichia coli* O157: H7, *Salmonella*, and *Shigella* for PulseNet. Foodborne Pathogens and Disease.

[ref-37] Sargeant J, Sanderson M, Smith R, Griffin D (2003). *Escherichia coli* O157: H7 in feedlot cattle feces and water in four major feeder-cattle states in the USA. Preventative Veterinary Medicine.

[ref-38] Sheng H, Shringi S, Baker K, Minnich S, Hovde C, Besser T (2016). Standardized *Escherichia coli* O157: H7 exposure studies in cattle provide evidence that bovine factors do not drive increased summertime colonization. Applied and Environmental Microbiology.

[ref-39] Shere J, Bartlett K, Kasper C (1998). Longitudinal study of *Escherichia coli* O157: H7 dissemination on four dairy farms in Wisconsin. Applied and Environmental Microbiology.

[ref-40] Stanford K, Agopsowicz C, McAllister T (2012). Genetic diversity and antimicrobial resistance among isolates of *Escherichia coli* O157: H7 from feces and hides of super-shedders and low-shedding pen-mates in two commercial beef feedlots. BMC Veterinary Research.

[ref-41] Teng L, Lee S, Park D, Jeong KC (2020). Genetic and functional analyses of virulence potential of an *Escherichia coli* O157: H7 strain isolated from super-shedder cattle. Frontiers in Cellular and Infection Microbiology.

[ref-42] Wells J, Berry E, Kim M, Bono J, Oliver W, Kalchayanand N, Wang R, Freetly H, Means W (2020). Determination of gastrointestinal tract colonization sites from feedlot cattle transiently shedding or super-shedding *Escherichia coli* O157: H7 at harvest. Journal of Applied Microbiology.

[ref-43] Williams M, Withee J, Ebel E, Bauer N, Schlosser W, Disney W, Smith D, Moxley R (2010). Determining the relationship between the seasonal occurrence of *Escherichia coli* O157: H7 in live cattle, ground beef, and humans. Foodborne Pathogens and Disease.

[ref-44] Xu Y, Dugat-Bony E, Zaheer R, Selinger L, Barbieri R, Munns K, McAllister T, Selinger B (2014). *Escherichia coli* O157: H7 super-shedder and non-shedder feedlot steers harbor distinct fecal bacterial communities. PLOS ONE.

