# Supplementary figures and images for: Understanding the transmission dynamics of Escherichia coli O157:H7 super-shedding infections in feedlot cattle
Source: PeerJ. 2021 Dec 20;9:e12524. doi: 10.7717/peerj.12524 (PMC8697766; doi:10.7717/peerj.12524)

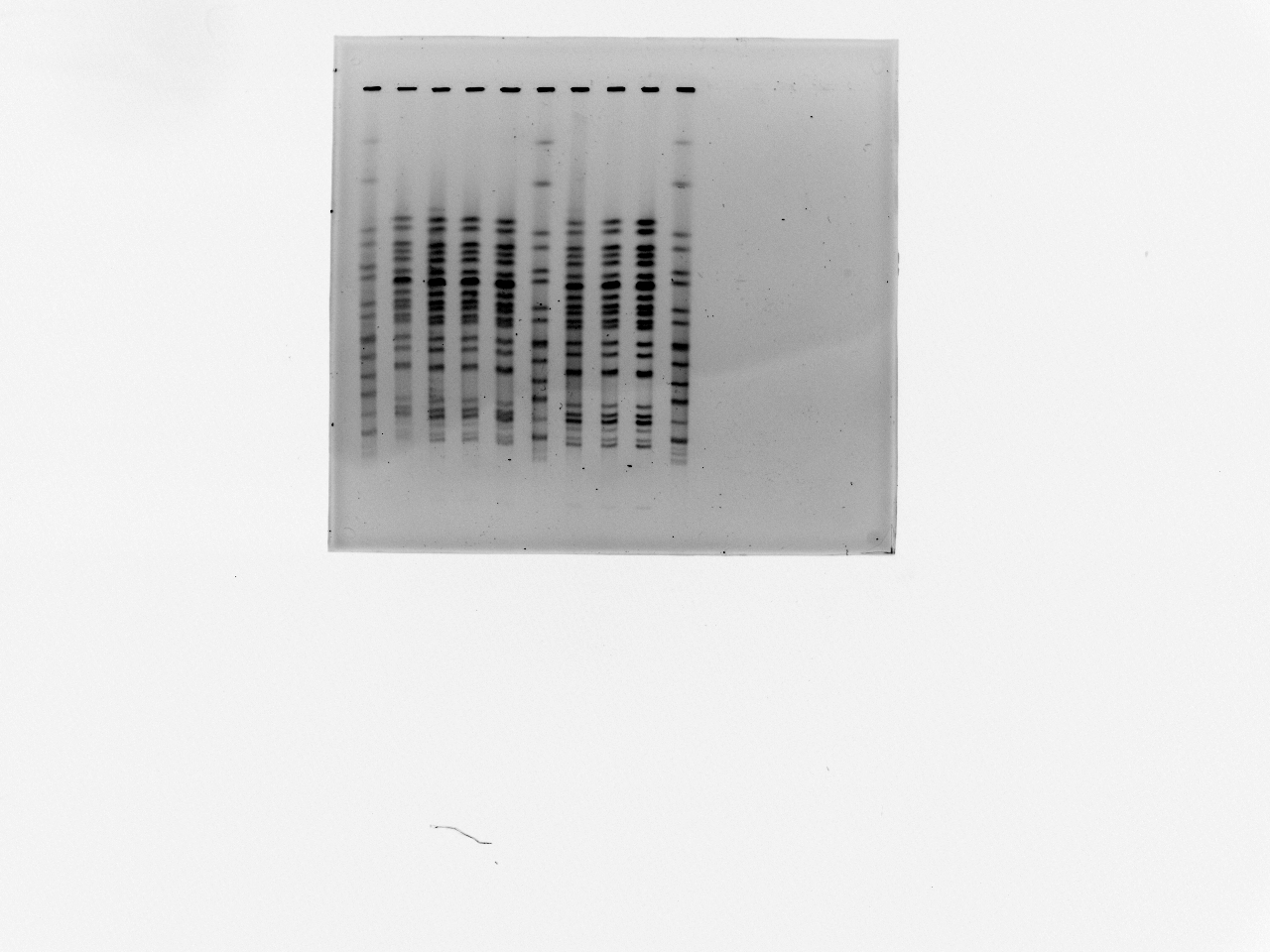

Supplement: Supplemental Information 5 — Raw gel images of bacterial fingerprint patterns for dendogram construction. [file peerj-09-12524-s005.zip › PFGE for A9 isolates in steer project 5-23-12.jpg]

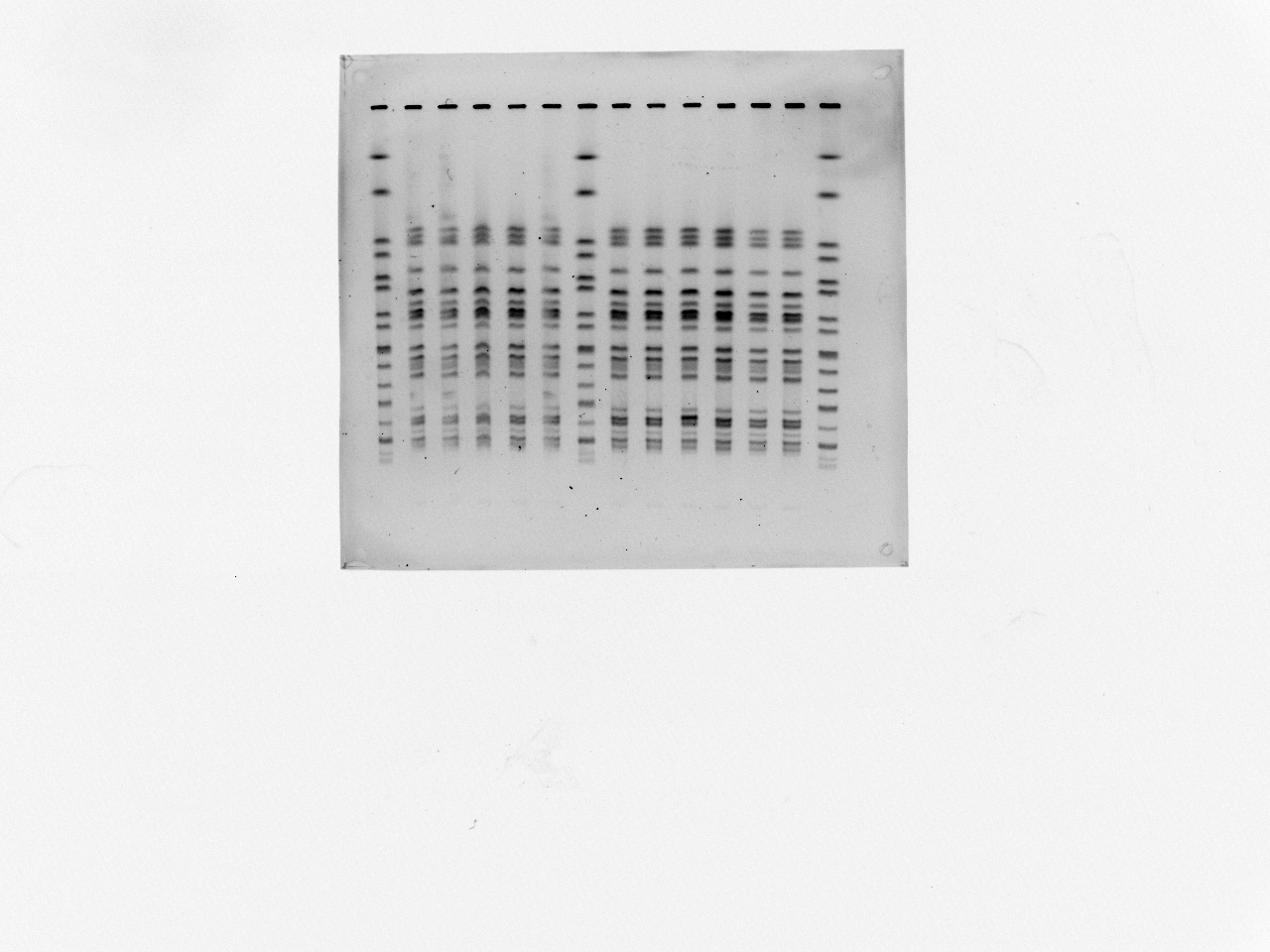

Supplement: Supplemental Information 5 — Raw gel images of bacterial fingerprint patterns for dendogram construction. [file peerj-09-12524-s005.zip › PFGE for Tx3800 isolates in steer project 6-17-12.jpg]

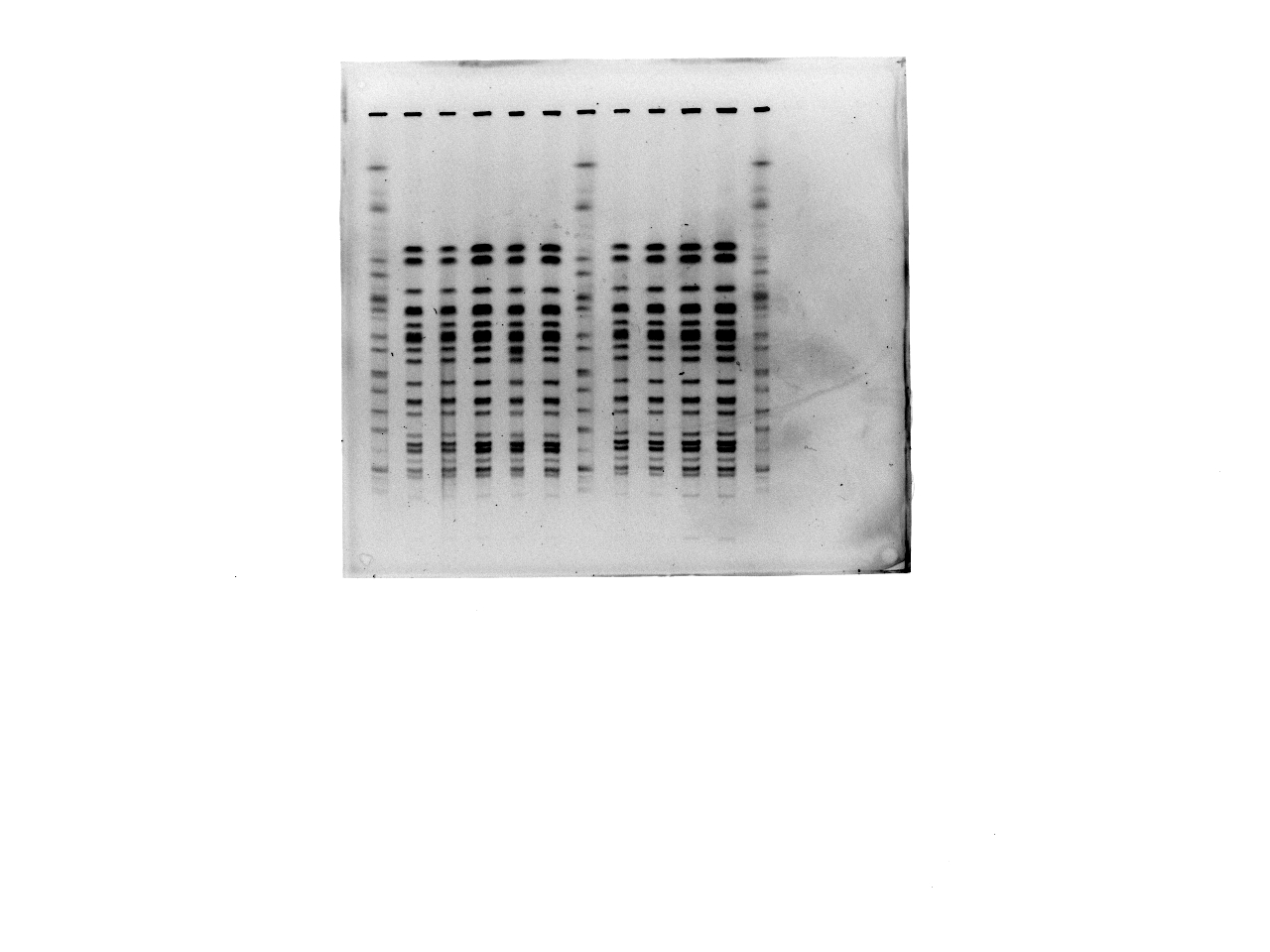

Supplement: Supplemental Information 5 — Raw gel images of bacterial fingerprint patterns for dendogram construction. [file peerj-09-12524-s005.zip › New PFGE gel for 14A isolates in steer project 6-6-12.jpg]

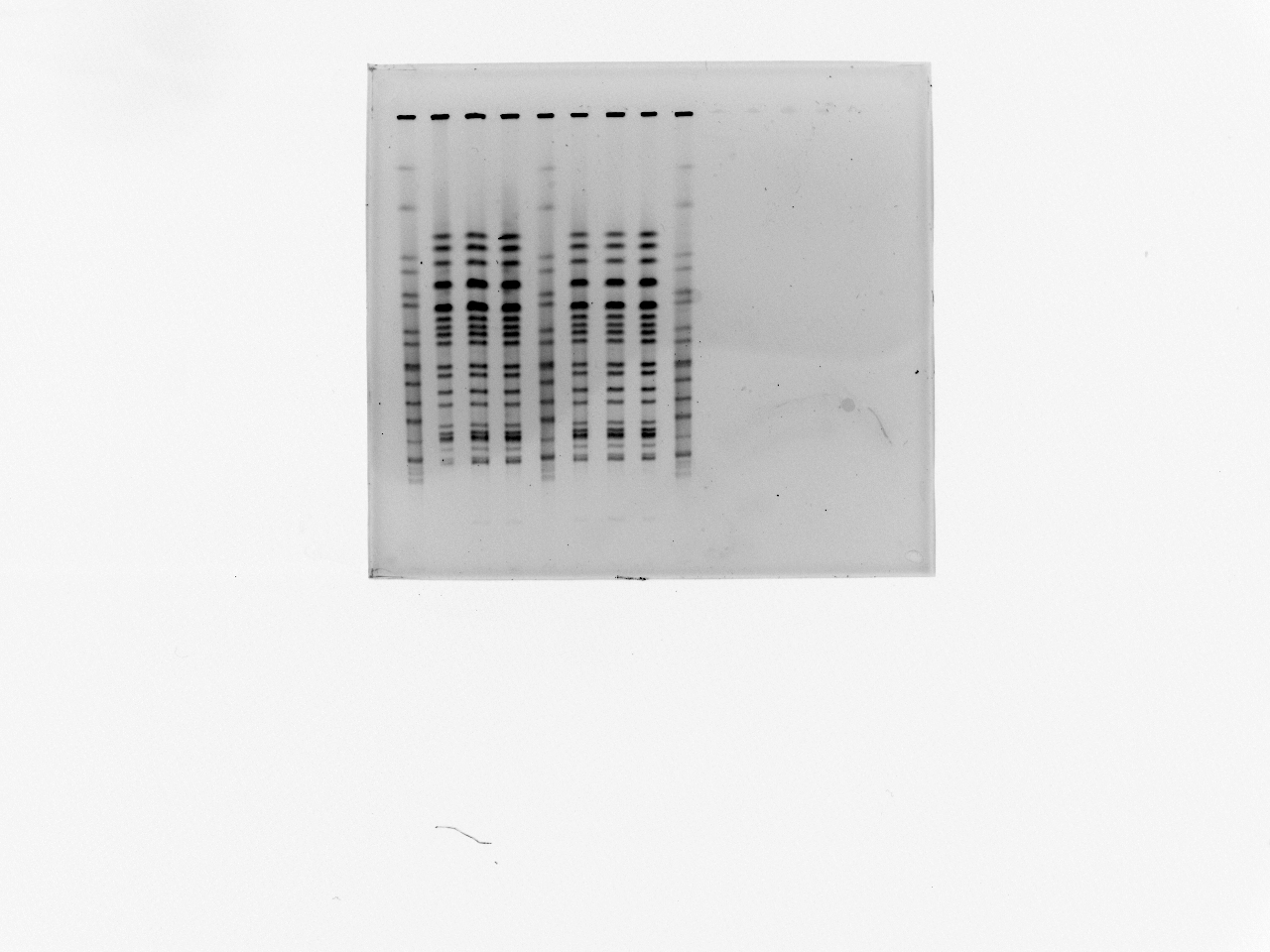

Supplement: Supplemental Information 5 — Raw gel images of bacterial fingerprint patterns for dendogram construction. [file peerj-09-12524-s005.zip › New PFGE gel for W1 isolates in steer project 5-25-12.jpg]

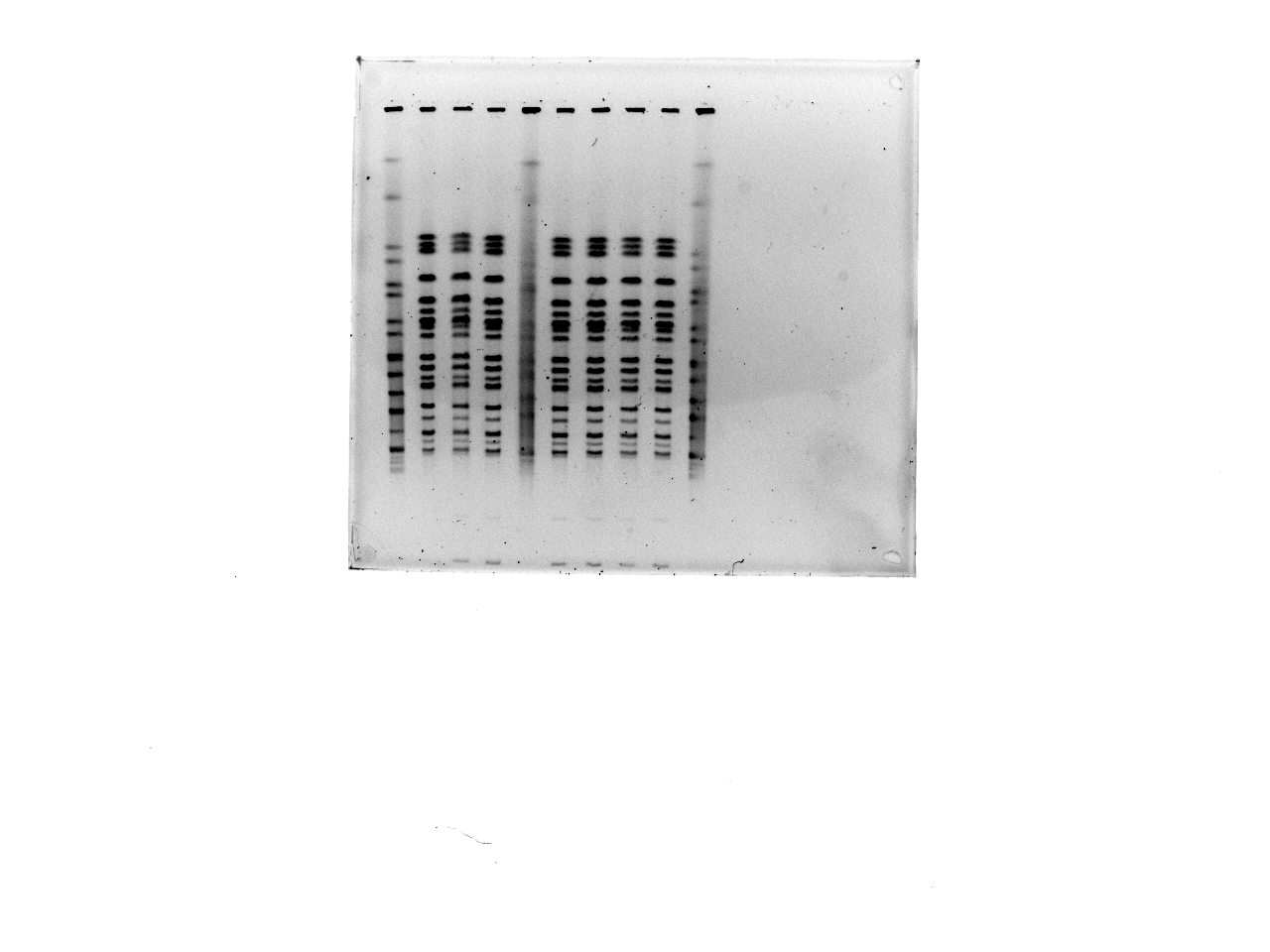

Supplement: Supplemental Information 5 — Raw gel images of bacterial fingerprint patterns for dendogram construction. [file peerj-09-12524-s005.zip › PFGE for #8055 isolates in steer project 5-29-12.jpg]

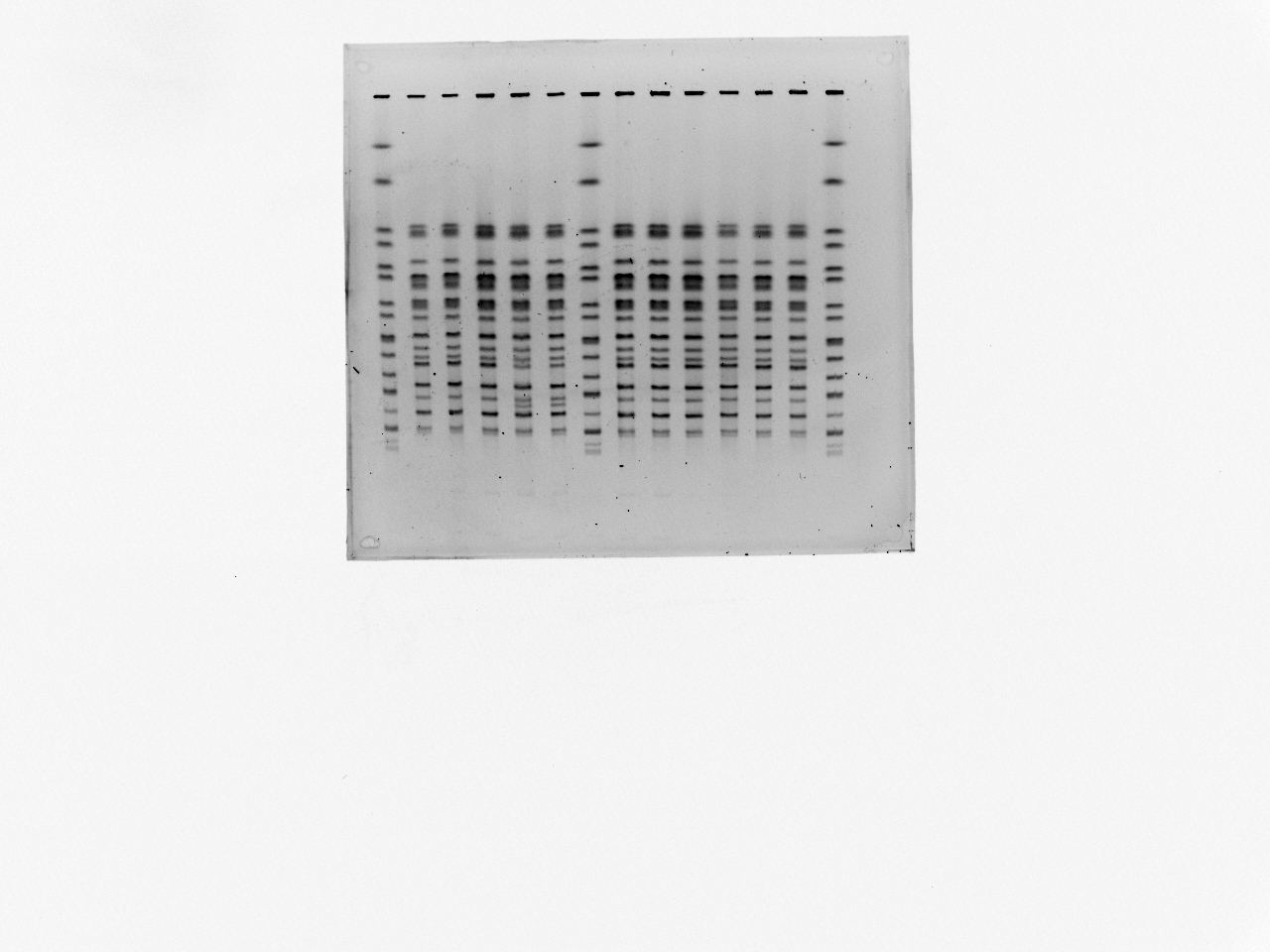

Supplement: Supplemental Information 5 — Raw gel images of bacterial fingerprint patterns for dendogram construction. [file peerj-09-12524-s005.zip › PFGE for Tx1686 isolates in steer project on 6-21-12, but needs to re-run again.jpg]
