# Supplementary material for: Understanding the transmission dynamics of Escherichia coli O157:H7 super-shedding infections in feedlot cattle
Source: PeerJ. 2021 Dec 20;9:e12524. doi: 10.7717/peerj.12524 (PMC8697766; doi:10.7717/peerj.12524)
Supplement: Supplemental Information 6 [file peerj-09-12524-s006.docx]

NCBA 2011 **Project**

**FEEDLOT DATA SHEET AND QUESTIONNAIRE**

___________Feedlot ID # ____________ Interviewer initials ______/_______/_____Sampling Date (mm/dd/yy)

Approx total precipitation during past month________(in) Average daily high/low temp past month______/______(F)

Which of the following are sources of water for the animals in this feedlot? (check all that apply)

☐ Pond, lake or other standing water ☐ Municipal or city

☐ Surface spring , stream, or river ☐ Well or ground water

| **0. Total cattle on feed on WHOLE feedlot #___________** | EARLY PEN #___________ | **LATE PEN #__________** |
| --- | --- | --- |
| 1. What is the ID the feedlot uses for this pen? |  |  |
| 2. How many beef steers are in this pen? |  |  |
| 3. How many beef heifers are in this pen? |  |  |
| 4. How many beef cows or bulls are in this pen? |  |  |
| 5. How many dairy steers are in this pen? |  |  |
| 6. How many dairy heifers/cows other than steers are in this pen? |  |  |
| 7. TOTAL number of cattle in pen (should equal sum of Q2 through Q7)? |  |  |
| 8. What is the average age in MONTHS of cattle in this pen ? | **________months** | **________months** |
| 9. What is the number of square feet in pen, or pen dimensions? | **_________sq ft or**  **_______ft. by ______ft.** | **_________sq ft or**  **______ft. by _____ft.** |
| 10. What date did the cattle in this pen arrive at the feedlot (mm/dd/yy) | **_____/_____/____** | **_____/_____/____** |
| 11. What was the initial average weight per head (in lbs) at placement? | **_________lbs** | **_________lbs** |
| 12. What is the current average weight per head (in lbs) in this pen? | **_________lbs** | **_________lbs** |
| 13. Is the pen under a full or partial cover or uncovered?  Circle correct choice for the pen. | **partial under cover**  **fully under cover**  **uncovered** | **partial under cover**  **fully under cover**  **uncovered** |
| 14. Is the watering location under a full or partial cover or uncovered?  Circle correct choice for the pen. | **partial under cover**  **fully under cover**  **uncovered** | **partial under cover**  **fully under cover**  **uncovered** |
| 15. Which of the following best describes the primary underlying surface of the pen? Circle correct choice for the pen. | **dirt**  **concrete**  **wooden slats** | **dirt**  **concrete**  **wooden slats** |
| 16. For how many days was this pen empty prior to placing cattle? | **__________days** | **__________days** |
| 17. Prior to the placement of the current cattle, was the manure in this pen scraped out or removed? | **y d/k n** | **y d/k n** |
| 18. If #17 was no, was the pen instead groomed or smoothed out before cattle were placed in the pen? If #17 was yes, skip to question 20. | **y d/k n** | **y d/k n** |
| 19. Approximately how many times has the pen been groomed or smoothed out while this group of cattle have been present in the pen? |  |  |
| 20. Is there a mound present in the pen? | **y n** | **y n** |
| 21. Approximately how many days has it been since the water trough or tank for this pen was last cleaned? Write NA if never cleaned; DK if don’t know | **_________days** | **_________days** |

**PAGE 2 NCBA 2011 Project**

|  | **EARLY PEN #___________** | LATE PEN #__________ |
| --- | --- | --- |
| 22. Which of the following methods were used to clean the water troughs or tanks for this pen for the last cleaning?  (See list on **List Sheet** below, circle all that apply, DK if don’t know) | **0 1 2**  **3 4**  **d/k** | **0 1 2**  **3 4**  **d/k** |
| 23. Where are the feed bunks located in the pen: **(a)** along a fenceline?  (Circle all that apply)  **(b)** inside the pen? | **y n**  **y n** | **y n**  **y n** |
| 24. How many days has it been since the feed bunk for this pen was cleaned? | **_________days** | **_________days** |
| 25. Which of the following methods were used to clean the feed bunks for this pen during the last cleaning?  (See list on **List Sheet** below, circle all that apply, DK if don’t know) | **0 1 2**  **3 4 5**  **d/k** | **0 1 2**  **3 4 5**  **d/k** |
| 26. Do you feed hay or mineral supplements (e.g. round bales, mineral blocks) off the floor of the pen? | **y d/k n** | **y d/k n** |
| 27. What is the percent concentrate that was fed to the cattle in this pen during the last 2 weeks?  How long have the cattle been on this % concentrate diet? | **__________%**  **__________days** | **__________%**  **__________days** |
| 28. Which of the following was the primary type of concentrate (i.e., largest % dry matter of concentrates) in the ration during the last 2 weeks? (See list on **List Sheet** below and circle choice, DK if don’t know) | **0 1 2**  **3 4 5**  **d/k** | **0 1 2**  **3 4 5**  **d/k** |
| 29. What is the major protein supplement in the concentrate?  **protein name**- | **_______________________** | **______________________** |
| 30. Which of the following was the major roughage (ie, the largest % dry matter of all roughages) fed to the cattle in this pen during the last 2 weeks? (See list on **List Sheet** below and circle correct choice, DK if don’t know) | **0 1 2 3**  **4 5 6 7**  **d/k** | **0 1 2 3**  **4 5 6 7**  **d/k** |
| 31. Were any ionophores fed to the cattle in this pen in the last 30 days? If yes, please write in the product names. **product name**- | **y d/k n**  **____________________** | **y d/k n**  **____________________** |
| 32. Were any other additives (e.g., probiotics, antibiotics) fed to the cattle in this pen in the last 30 days? Please write in the product names.  **product name**- | **y d/k n**  **____________________** | **y d/k n**  **____________________** |
| 33. Did the cattle in this pen come from: **(a)** a single source?  **(b)** multiple sources? | **y n**  **y n** | **y n**  **y n** |
| 34. Which of the following sources describe where the cattle in this pen came from? (See list on **List Sheet** below and circle all that apply, DK if don’t know) | **0 1 2 3**  **4 5 6**  **d/k** | **0 1 2 3**  **4 5 6**  **d/k** |
| 35. How many cases of **diarrhea** or **respiratory disease** in this pen during last month? | **_______________# diarrhea**  **______________# respiratory** | **_____________# diarrhea**  **___________# respiratory** |
| 36. How many cattle have **died** in this pen from day of arrival? | **__________________# dead** | **_________________# dead** |

**PAGE 3**

**NCBA 2011 Project**

**LIST SHEET**

**# 22 Water trough/tank cleaning methods**

0 nothing done; not cleaned

1 trough/tank drained of water

2 trough/tank scrubbed

3 chemically disinfected

4 other method used

**# 25 Feed bunk cleaning methods**

0 nothing done; not cleaned

1 bunk is swept out by hand or rotary broom

2 bunk is blown out with tractor mounted blower

3 bunk is shoveled out

4 bunk is treated with disinfectant

5 other method used

**# 28 Major type of energy concentrate in ration**

0 none fed

1 corn or corn by-products

2 wheat or wheat by-products

3 barley

4 milo

5 other type

**# 30 Major type of roughage in ration**

0 none fed

1 alfalfa hay

2 grass hay (sudan, timothy, native, etc)

3 cereal hay (seed head is on)

4 cereal straw (seed head removed)

5 agricultural byproducts (beet pulp, nut hulls, etc)

6 corn silage

7 other type

**# 34 Sources of cattle**

0 direct from a single farm or ranch where the cattle were on *pasture or range*

1 direct from multiple farms or ranches where the cattle were on *pasture or range*

2 direct from a single backgrounding operation where the cattle were on *drylot*

3 direct from multiple backgrounding operations where the cattle were on *drylots*

4 auction market-single owner

5 auction market-multiple owners

6 other
